# Sensitive, specific, and rapid on-site detection of calf diarrhea pathogens using the RPA-CRISPR/Cas 12a assay

**DOI:** 10.3389/fcimb.2026.1734185

**Published:** 2026-02-02

**Authors:** Yan Wang, Yun Diao, Tianqi Zhang, Fan Zhang, Wei Wang

**Affiliations:** 1 State Key Laboratory of Reproductive Regulation and Breeding of Grassland Livestock, College of Life Sciences, Inner Mongolia University, Hohhot, China; 2 R&D Department Inner Mongolia Mengwei Biotech Co., Ltd, Hohhot, China; 3 State Key Laboratory of Animal Disease Control and Prevention, Lanzhou Veterinary Research Institute, Chinese Academy of Agricultural Sciences, Lanzhou, China

**Keywords:** BCoV, BRV, BVDV, ETEC, real-time qPCR assay, RPA-CRISPR/Cas12a assay

## Abstract

Calf diarrhea is a common gastrointestinal disease that usually occurs within one month of birth. The disease causes the greatest economic losses to the cattle industry. Currently, a variety of diagnostic methods have been developed for calf diarrhea infections. However, existing methods are still unsatisfactory in terms of sensitivity, specificity, simplicity, cost, and speed.To provide a more sensitive, specific, simpler, and faster detection method, we recently developed an RPA-CRISPR/Cas12a assay that can detect BVDV, BCoV, BRV, and ETEC infections in cattle on-site. Testing for each pathogen is performed in a single test tube, without the need to open the tube in the middle, and can be completed in under 50 minutes.The RPA-CRISPR/Cas12a assay can detect BVDV, BCoV, BRV, and ETEC at concentrations of at least 10 copies/μL. The RPA-CRISPR/Cas12a assay does not produce false-positive results due to the presence of other pathogens. The sensitivity of BCoV, BRV, and ETEC in the RPA-CRISPR/Cas12a quadruple assay is equivalent to that of single qPCR. The sensitivity of BVDV in the quadruple assay is slightly lower than that of the single qPCR method.Due to its sensitivity, specificity, simplicity, and rapidity, the RPA-CRISPR/Cas12a assay is more practical for on-site detection of cattle diarrhea pathogens than any existing detection method.

## Introduction

Calf diarrhea, also known as calf scour, is a common gastrointestinal disease in calves that usually develops within the first month of life, especially in the first two to three weeks of life. While cattle of any age can develop diarrhea, young calves are particularly susceptible. The disease poses a threat to the healthy development of the global cattle industry and causes significant economic losses to cattle farms worldwide. The leading cause of diarrhea is usually the invasion of the intestines by pathogens, including viruses, bacteria, and parasites, including bovine rotavirus (BRV), bovine coronavirus (BCoV), bovine viral diarrhea virus (BVDV), *Salmonella* spp. (S. spp.), *Escherichia coli* (ETEC), *Clostridium perfringens* (C. perfringens), and *Cryptosporidium parvum* (C. parvum) along with newly emerging enteric pathogens such as bovine torovirus (BToV) and caliciviruses (bovine norovirus [BNoV] and Nebovirus) (Cho and Yoon, 2014).

Clinical manifestations include neonatal calf diarrhea, characterized by stools that range from watery to brown, gray, yellow, or green. Sometimes blood and mucus may appear in the stool. Other nonspecific symptoms include loss of appetite, malaise, unsteadiness when walking, and weakness in standing. Severe cases may cause depression, dehydration, hypoglycemia, and/or disturbances in body fluid and acid-base balance. Severe dehydration and electrolyte imbalance can be fatal if not treated promptly, usually leading to rapid death (NAHMS US, 2007). The global prevalence of BRV is approximately 33.7% (Papp et al., 2013). The prevalence of BCoV in European countries ranges from 2.80% to 46.74% (Geng et al., 2023). The antigen prevalence of BVDV is as high as 15.74% (Su et al., 2023). The prevalence of diarrhea in dairy calves varies from 38.5% in the USA, 23% in Canada, 53% in Korea, and 27.2% in China (Gomez and Weese, 2017; Kim et al., 2021; Qu et al., 2023; Urie et al., 2018; Windeyer et al., 2014).

Currently, a variety of diagnostic methods have been developed for calf diarrhea infections. The traditional methods for detecting calf diarrhea viruses typically have included pathogen isolation and characterization, detection of antigens and antibodies, along with histopathology, mass spectrometric analysis, ELISA, fluorescence polarization, histopathological analysis, dot blot (Xing et al., 2009), real-time fluorescence quantitative PCR (Rousselon et al., 2004), and DNA microarray (Kim et al., 2007). The disadvantages of these techniques are that they are time-consuming, require special instruments, and are difficult to test large numbers of samples in the field. In 2006, recombinase polymerase amplification (RPA), A sensitive isothermal molecular diagnostic technology, became the molecular tool of choice for rapid, specific, and cost-effective identification of pathogens (Daher et al., 2016). Later, isothermal amplification technology, RPA, and clustered regularly interspaced short palindromic repeats (CRISPR)/Cas12a were combined for detection without compromising the sensitivity and specificity of molecular detection. CRISPR and CRISPR-associated (Cas) protein systems can recognize and cleave specific nucleic acid sequences. Target recognition by crRNAs directs the silencing of the foreign sequences through Cas proteins that function in complex with the crRNAs. Based on this property, they can convert the sequence information of target nucleic acids into visual readout signals, such as fluorescence and electrochemical values. Pre-amplification can further improve the system’s sensitivity (Platt et al., 2014). Nowadays, portable detection methods based on the CRISPR/Cas system are widely used.

We recently developed an RPA-CRISPR/Cas12a assay for detecting BVDV, BCoV, BRV, and ETEC pathogens in fecal samples of diarrheal cattle. This detection method has the distinct advantages of high sensitivity, strong specificity, simplicity, rapidity, and is very useful for on-site diagnosis. Here, we report our results as follows.

## 2 Materials and methods

### 2.1 Sample collection, recombinant plasmid preparation, and DNA extraction

Fecal swab samples were collected from 59 diarrheic cattle. RNA was extracted from these samples according to the instructions of the M5 Viral DNA/RNA Kit (Mei5 Biotechnology Co., Ltd., Beijing, China). The RNA samples were reverse transcribed into cDNA using the “Sui Liu” ultra-fast reverse transcription kit (Mei5 Biotechnology Co., Ltd, Beijing, China) and stored in a -20°C refrigerator for later use.

Obtain gene sequences for BVDV, BCoV, BRV, and ETEC from the NCBI database (http://www.ncbi.nlm.nih.gov/). MAGA7 sequences of four pathogens were aligned to obtain conserved region sequencesaaa (**Supplementary Figure S1**). Conservative sequences of 100 to 300 bp were screened in the BVDV 5’-UTR region, BCoV N region, BRV VP7 region, and ETEC STa region. To prepare DNA standard templates, four conserved genomic sequences of calf diarrhea pathogens (BVDV 201 bp, BCoV 222 bp, BRV 135 bp, ETEC 175 bp) were cloned into the pUC57 vector (Liuhe BGI Genomics Co., Ltd., Beijing). *E. coli* (*DH5α* )cultures containing recombinant plasmid DNA were grown overnight at 37°C and 220 rpm on a shaker. DNA was extracted according to the instructions of the Zhejiang EasySide Plasmid DNA Micro Extraction Kit (Zhejiang EasySide Biotechnology Co., Ltd., Zhejiang, China), and the recombinant plasmids pUC57-BVDV, pUC57-BCoV, pUC57-BRV, and PUC57-ETEC were stored in a -20°C refrigerator for future use.

### 2.2 Design and synthesis of primers, crRNA, and ssDNA reporter

Primers were designed in the conserved regions of each pathogen according to the primer design guidelines provided by TwistDx. Primers were synthesized and purified by Sangon Biotech (Shanghai) Co., Ltd. (Shanghai, China). A 23 bp crRNA was designed in the conserved region, and a scaffold sequence was added to the front end of the crRNA to help the Cas 12a protein activate the cutting activity. The ssDNA reporter probe sequence was designed as 5’-FAM-TTATT-BHQ1-3’. Both crRNA and ssDNA reporters were synthesized and purified by Sangon Biotech (Shanghai) Co., Ltd. (Shanghai, China) (**Table 1**).

**Table 1:** Sequences of primers, crRNA, and ssDNA reporter.

Assay	Oligonucleotide sequences	Sequence (5’-3’)
RPA	BVDV F1	GCAGTTCTAACCGACTGTTACGAATACAGCCTGAT
	BVDV F2	TAGTCGTCAGTGGTTCGACGCCTTGGAATA
	BVDV F3	CAGTGGTTCGACGCCTTGGAATAAAGGTCTC
	BVDV R1	ACAACTCCATGTGCCATGTACAGCAGAGATT
	BVDV R2	GTGCCATGTACAGCAGAGATTTTTAGTAGC
	BVDV R3	CCATGTACAGCAGAGATTTTTAGTAGCAATAC
	BCoV F1	GAGTTTGAATTTGCAGAGGGACAAGGTGTGCC
	BCoV F2	GTTACTATATTGAAGGCTCAGGAAGGTCTGC
	BCoV F3	GACAAGGTGTGCCTATTGCACCAGGAGTCCCAGC
	BCoV R1	AAACCTAGTCGGAATAGCCTCATCGCTACTTG
	BCoV R2	CAGTCTGCTTAGTTACTTGCTGTGGCTTAG
	BCoV R3	GTATTGACATCAGCCTGGTTACTAGCGACCCAG
	BRV F1	AGCGACTACATGGTACTTCAATCCAGTGAT
	BRV F2	CCTTTGTTTGTATTATCCTGTTGAGGCATC
	BRV F3	GCCAACAGGATCAGTGTACCTTAAAGAATA
	BRV R1	ACATCTGTAATCACTAACTTCTCCGTTGTC
	BRV R2	GCCAATTCAGACATATCTAGTTCTTGTGTAGAGTC
	BRV R3	GCAGATTCAATTCTAAGCGTGAGTCCTACT
	ETEC F1	GGGATCCGGATCCGGATCGGCGCGCCGCGATCG
	ETEC F2	GATCATGATCATGATCGCGGCCGCCCTGGC
	ETEC F3	GCGAGTGTACCTCGACATATAACATGATGC
	ETEC R1	TTAATAACATCCAGCACAGGCAGGATTACA
	ETEC R2	TTTCTGTATTATCTTTCCCCTCTTTTAGTCAGTCA
	ETEC R3	CCATATGATGCATGAATTCCAGCAGCTGCTG
Cas12a assay	BVDV crRNA1	UAAUUUCUACCCCCCGUAGAUGGAUGGCCGAACCCCUGAGUACAGGG
	BVDV crRNA2	UAAUUUCUACCCCCCGUAGAUUUCGUUGGAUGGCUUAAGCCCUGA
	BVDV crRNA3	UAAUUUCUACCCCCCGUAGAUGUAGCAGAACAGUGGGCCUCUGC
	BCoV crRNA1	UAAUUUCUACCCCCCGUAGAUGCCAGAACAAGACUAGCAAUUUG
	BCoV crRNA2	UAAUUUCUACCCCCCGUAGAUAUCAGCCAUAUCAGGUGUUACAC
	BCoV crRNA3	UAAUUUCUACCCCCCGUAGAUGCAGUCUGCUUAGUUACUUGCUG
	BRV crRNA1	UAAUUUCUACCCCCCGUAGAUAGUGGAACCACAGUUAUACUGC
	BRV crRNA2	UAAUUUCUACCCCCCGUAGAUAUGCCUCAACAGGAUAAUACAAA
	BRV crRNA3	UAAUUUCUACCCCCCGUAGAUGUUUGAUGCCUCAACAGGAUAAU
	ETEC crRNA1	UAAUUUCUACCCCCCGUAGAUUCAGCACCAAUACAUAUAAUAUA
	ETEC crRNA2	UAAUUUCUACCCCCCGUAGAUAUGUUACCUCCCGUCAUGUUGUU
	ETEC crRNA3	UAAUUUCUACCCCCCGUAGAUAAGGCGGAUCCGCCGAGCUCGAU
	ssDNA reporter	FAM-CCGGAAAAAAAAAAAACCGG-BHQ1
Real-time qPCR	BVDV	GCAGTTCTAACCGACTGTTACGAATACAGCCTGAT GTGCCATGTACAGCAGAGATTTTTAGTAGC
	BCoV	ATATTGAAGGCTCAGGAAGG TAGTTACTTGCTGTGGCTTA
	BRV	CCAACAGGATCAGTGTACCTTAAAGAATA GCCAATTCAGACATATCTAGTTCTTGTGTAGAGTC
	ETEC	GAGTGTACCTCGACATATAAC AGAGTCAAGTGATTCAGTTG

### 2.3 Establishment and optimization of the RPA-CRISPR/Cas12a assay

RPA reactions were performed in 50 μL volumes using the commercial TwistAmp^®^ Basic kit (TwistDx Inc., UK) at the recommended temperature of 39°C. Briefly, a reaction mixture consisting of 29.5μL of reaction buffer, 1.5 μL of forward primer (10 μM) and reverse primer (10 μM) for each of the four pathogens, and 2 μL of diethylpyrocarbonate (DEPC)-treated water was added to the tube containing the lyophilized RPA enzyme mix. Then, add 1μL (100ng/ul) of each of the four-pathogen template DNAs and 2.5 μL of magnesium acetate, and incubate at 39°C for 20 min. The RPA product was verified by 2% agarose gel electrophoresis. Then, the RPA amplification products were added to 20 μL CRISPR Cas12a systems and reacted at 39°C for 30 minutes. Of which, the reaction mixture consists of 2 μL NEB Buffer 2.1, 1 μL LbaCas12a, 1μL ssDNA Reporter, 1μL crRNA, 13 μL DEPC-treated water, and 2 μL RPA products. Use a fluorescence quantitative PCR instrument (Analytikjena Inc., Germany) to read and record the fluorescence signal of the reaction every minute. After the reaction, the sample tube is placed under ultraviolet and/or blue light (470 nm) to observe the fluorescence signal. Then, the concentration ratios of primers RPA BVDV, BCoV, BRV, ETEC, ssDNA Reporter, cRNA, and Cas12a were optimized in the RPA-CRISPR/Cas12a assay. The ssDNA Reporter was set at four concentration gradients of 200 nM/L, 300 nM/L, 400 M/L and 500 nM/L. The total volumes for the CRISPR Cas12a reaction systems are 10, 20, 30, 40, and 50 μL respectively. The crRNA was set at three concentration gradients of 0.5 uM, 1 uM, and 1.5 uM, and the Cas12a was set at three concentration gradients of 40 nM, 50 nM and 60 nM.

### 2.4 Sensitivity of RPA-CRISPR/Cas12a

To evaluate the assay sensitivity, serial dilutions of pUC-BVDV, pUC57-BCoV, pUC57-BRV, and pUC57-ETEC as templates were quantified using a NanoDrop 2000 spectrophotometer (Thermo Fisher Scientific, MA, USA) and tested by the RPA-CRISPR/Cas12a assay at a concentration gradient from 10^5^, 10^4^, 10^3^, 10^2^, and 10 copies/μL, respectively. Bovine parainfluenza virus type 3 (BPIV-3), bovine herpesvirus type 4 (BoHV-4), and *E. coli* strain DH5a were used as negative controls. The test procedure was the same as that described in 2.3 above. The results were read under blue light or UV light. Sensitivity results were defined as the highest dilution of plasmid DNA.

### 2.5 Detection of calf fecal samples

The RNA extracted from calf feces samples was reverse transcribed into cDNA. These cDNA products were then used in RPA-CRISPR/Cas12a assays. Primers specific for each pathogen (BVDV F2 R2, BCoV F2 R2, BRV F3 R2, ETEC F3 R2) were used in quadruple RPA reactions described in 2.3. Brierly, the reaction was carried out for 20 minutes at 39°C in a metal bath (Dino Direct China Limited). Then, the RPA reaction product was used in the CRISPR/Cas12a assay, and the reaction was carried out in a bath at 39°C for 30 min. At the end of the reaction, the tubes were irradiated with a blue light, and the reaction tubes were labeled positive if they emitted a yellowish-green fluorescence and negative if they did not emit light. If the luminescent signal be unclear, supplementary analysis using ImageJ may be carried out. Determine whether the result is negative or positive based on the Mean grayscale value measured in the emitting region of each RCR tubule. DNA from 59 calf fecal samples was also analyzed using real-time qPCR assay detection procedures. The reaction volumes of the real-time qPCR assay were 20 μL with 10 μL of TB Green (Takara Biomedical Technology (Beijing) Co., Ltd., Beijing, China), 1 μL of forward primer, 1 μL of reverse primer, 2 μL of template, and 6 μL of DEPC water. Thermocycling was performed in a Real-time qPCR system (Analytik Jena AG, Germany) at the following conditions: 95°C for 30 s, one cycle; 95°C for 5 s, 60°C for 20 s, 40 cycles, and 95°C for 10 s, 65°C for 15 s, one cycle. The relative threshold (Crt) value is used to determine the cycle at which a sample is considered positive.

## 3 Results

### 3.1 RPA-CRISPR/Cas12a assay optimization

**Figure 1:**
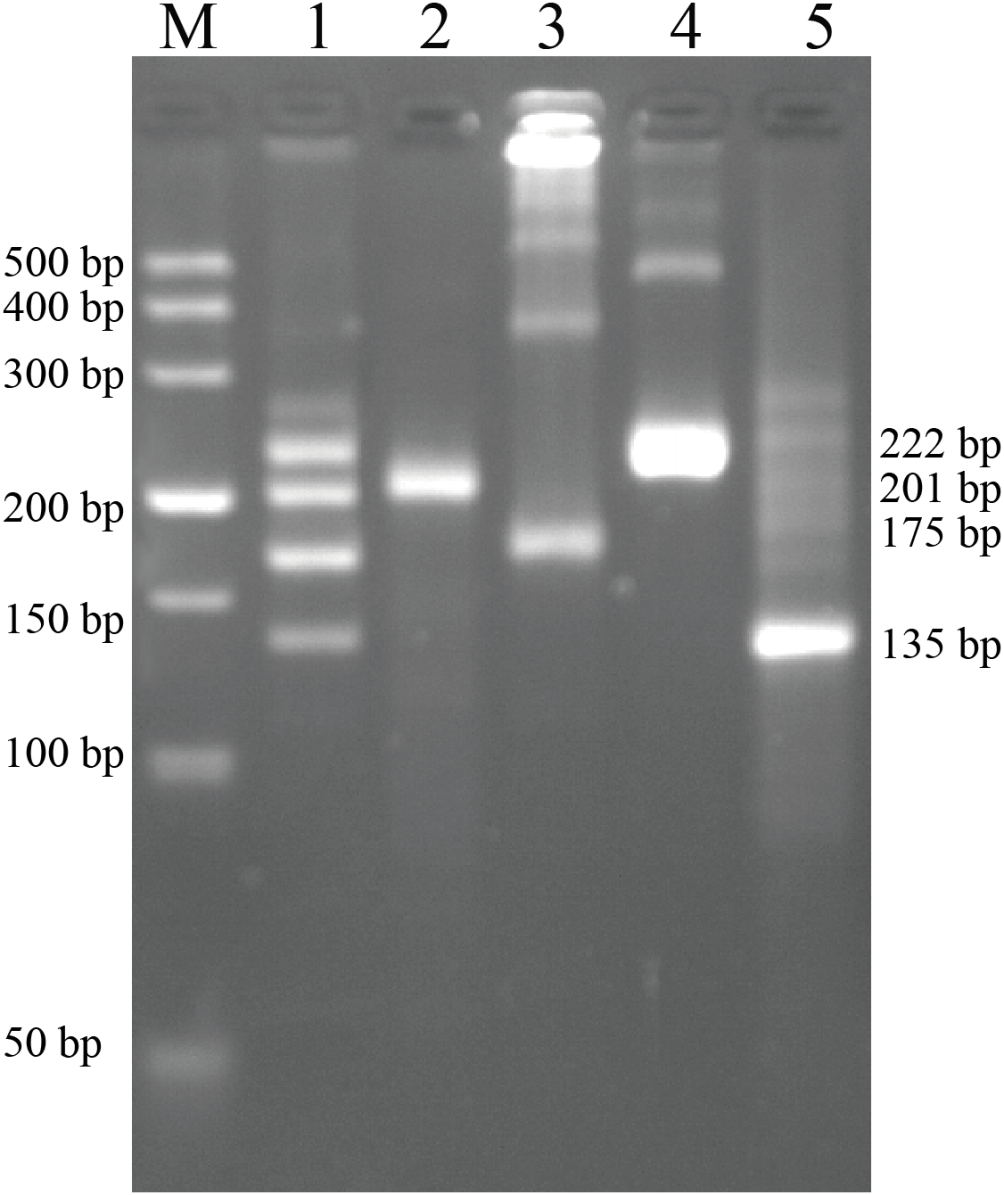
Design of primers for specific detection of calf pathogens. Lane 1: Templates for four pathogens and optimal primer pairs for each; Lane 2-Lane 5: Each containing only the single template of BVDV (201 bp), ETEC (175 bp), BCoV (222 bp), or BRV (135 bp) together with the corresponding primer pair.

Our first goal was to test the ability of the designed primers to detect pathogens and differentiate between pathogens in calves. First, the optimal primers for individual pathogens were screened (**Supplementary Figure S1**). Subsequently, the recombinant plasmids of pUC57-BVDV, pUC57-BCoV, pUC57-BRV, and pUC57-ETEC were tested in the RPA amplifications. The products were initially validated by 2% (w/v) agarose gel electrophoresis. The results showed that the designed primers were able to specifically distinguish four different calf pathogens, indicating that the primers were reliable and had no cross-reaction. The results of an agarose gel electrophoresis are shown in **Figure 1**. Based on this, we optimized the RPA-CRISPR/Cas12a system. The optimal reaction volume for the CRISPR–Cas12a assay was 20 μL (**Supplementary Figure S3**). For the four target pathogens, the best-performing crRNAs were BDVD crRNA3, BCoV crRNA2, BRV crRNA2, and ETEC crRNA2 (**Supplementary Figure S4**). Balancing assay performance and cost, a single-stranded DNA (ssDNA) fluorescent reporter concentration of 500 nM was chosen as optimal (**Supplementary Figure S5**). The optimal concentrations were 50 nM Lba Cas12a and 0.5 μM crRNA (**Supplementary Figure S6**). We conducted subsequent experiments using the optimal system.

To verify the roles of different components in the RPA-CRISPR/Cas12a assay, we analyzed different combinations (**Table 2**, **Supplementary Figure S7**). A fluorescence intensity greater than 5000 is defined as positive and the results showed that the lack of any reaction component in the reaction system would lead to a negative reaction result. This result shows that a correct positive reaction can only be obtained if all reaction components are present. No false-positive reactions were observed in the assay.

**Table 2:** Analyzing the impact of each component in the RPA-CRISPR/Cas12a assay.

Components\Tube No.	1	2	3	4	5	6
NEB Buffer 2.1	–	+	+	+	+	+
Lba Cas12a	+	–	+	+	+	+
crRNA	+	+	–	+	+	+
ssDNA reporter	+	+	+	–	+	+
Template	+	+	+	+	–	+
Primers	+	+	+	+	+	+
DEPC Water	+	+	+	+	+	+

### 3.2 Sensitivity of the RPA-CRISPR/Cas12a-fluorescence assay

**Figure 2:**
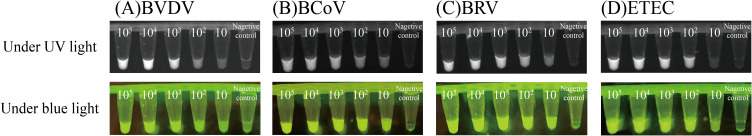
Sensitivity of RPA-CRISPR/Cas12a fluorescence detection. Under UV and blue light irradiation, the fluorescence signal intensity of **(A)** BVDV, **(B)** BCoV, **(C)** BRV, and **(D)** ETEC increased from 10 copies/μL to 10^5^ copies/μL.

To evaluate the assay sensitivity, serial dilutions of pUC-BVDV, pUC57-BCoV, pUC57-BRV, and pUC57-ETEC as templates were tested by the CRISPR/Cas12a assay at a concentration gradient from 10^5^, 10^4^, 10^3^, 10^2^, and 10 copies/μL, respectively. This study was conducted in triplicate, with fluorescence intensity greater than 5000 defined as positive. The test results showed that RPA-CRISPR/Cas12a-assay could detect at least 10 copies/μL of pathogen DNA (**Figure 2**, **Supplementary Figure S8**).

### 3.3 Specificity of the RPA-CRISPR/Cas12a assay

**Figure 3:**
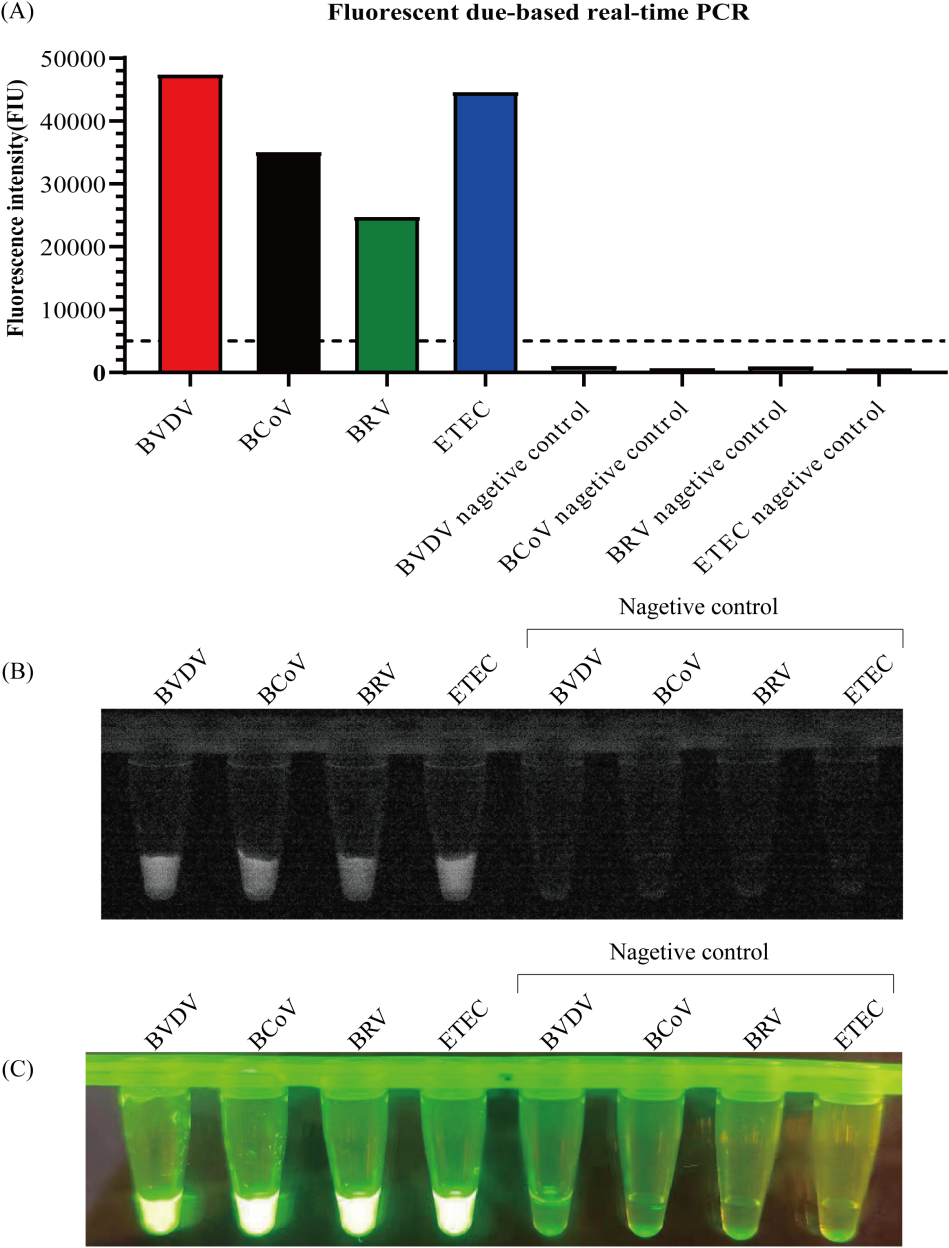
Specificity of RPA-CRISPR/Cas12a for detecting plasmids containing DNA of calf diarrhea pathogens. **(A)** The graph shows the fluorescence intensity of plasmids containing four different calf pathogen plasmid DNA and pathogen-free plasmids tested by real-time qPCR. **(B, C)** Plasmid DNA containing four different calf pathogens and those without pathogens were examined under UV and blue light tested by RPA-CRISPR/Cas12a assay.

Validation experiments were performed using plasmids containing DNA of four different calf pathogens and plasmids without pathogen DNA. The fluorescence intensity results of the RPA-CRISPR/Cas12a assay and qPCR of four calf pathogens showed that there were obvious differences between positive and negative samples (**Figure 3**, **Supplementary Figure S9**).

**Figure 4:**
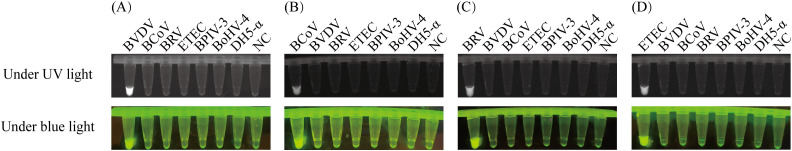
Specificity of the RPA-CRISPR/Cas12a assay for detecting calf diarrhea pathogens under UV light and blue light. **(A)** Tube 1 contains the plasmid of BVDV DNA, and the other tubes are negative controls. **(B)** Tube 1 contains the plasmid of BCov DNA, and the other tubes are negative controls. **(C)** Tube 1 contains the plasmid of BRV DNA, and the other tubes are negative controls. **(D)** Tube 1 contains the plasmid of ETEC DNA, and the other tubes are negative controls.

To evaluate the specificity of the RPA-CRISPR/Cas12a assay, the fluorescence signal generated by the target DNA sequence should also be compared with that generated by non-target DNA sequences. In this study, plasmids containing four different calf diarrhea pathogens, BVDV, BCoV, BRV, and ETEC, and negative controls, BPIV-3, BoHV-4, and DH5a, were tested. Our result shows that high specificity means that the target emits a strong fluorescent signal, while various negative controls have no fluorescent signal (**Figure 4**, **Supplementary Figure S10**).

### 3.4 Comparison of RPA-CRISPR/Cas12a assay and real-time qPCR assay for detecting calf fecal pathogens

In this study, fecal samples from 59 calves were tested in parallel by RPA–CRISPR/Cas12a assay and qPCR assay. Fluorescence from the RPA–CRISPR/Cas12a assay was quantified with ImageJ, and samples with a Mean gray value (GV) > 40 were classified as positive; all others were negative. qPCR assay results were defined as positive when fluorescence ≥ 10000 and Ct < 33, with all other results considered negative. By these criteria, the RPA–CRISPR/Cas12a positive rates were 15.3% (9/59) for BVDV, 1.7% (1/59) for BCoV, 37.3% (22/59) for BRV, and 28.8% (17/59) for ETEC (**Supplementary Tables S1**-**S4**; **Supplementary Figure S11**); the corresponding qPCR positive rates were 18.6% (11/59), 1.7% (1/59), 37.3% (22/59), and 28.8% (17/59) (**Table 3**, **Supplementary Figure S12**). Using qPCR as the reference standard, the RPA-CRISPR/Cas12a assay demonstrated a sensitivity of 81.8% (95% CI: 52.3–95.1%) for BVDV, and 100% for BCoV, BRV, and ETEC (95% CIs: 59.0–100.0%, 88.1–100.0%, and 84.7–100.0%, respectively). Specificity was 100% for all tested pathogens (95% CIs ranging from 90.5% to 100.0%). Furthermore, the assay showed high concordance with the reference method (Kappa values: 0.88–1.00) (**Supplementary Table S5**). The detection result and mixed infection patterns of the two methods in 59 fecal swabs are shown in **Supplementary Figure S13**.

**Table 3:** Comparison of RPA-CRISPR/Cas12a assay and real-time qPCR assay for detecting calf fecal pathogens.

Assays pathogens	RPA-CRISPR/Cas12a assay		Real-time qPCR assay		Accordance rate (%)
	Positive sample/Total sample	Positive rate (%)	Positive sample /Total samples	Positive rate (%)	
BVDV	9/59	15.3	11/59	18.6	82
BCoV	1/59	1.7	1/59	1.7	100
BRV	22/59	37.3	22/59	37.3	100
ETEC	17/59	28.8	17/59	28.8	100

## 4 Discussion

Neonatal calf diarrhea is usually characterized by rapid onset and high mortality. Sensitive, specific, and easy on-site pathogen identification is essential for prevention and treatment. However, existing nucleic acid detection methods are still unsatisfactory (Gootenberg et al., 2017). In this perspective, we recently developed and demonstrated that the RPA-CRISPR/Cas12a assay performs well in detecting BVDV, BCoV, BRV, and ETEC infections in calf fecal samples. In 2012, Craw and Balachandran, and other researchers reported that the RPA assay could amplify specific DNA sequences at constant low temperatures, making it a versatile alternative to PCR. It uses a recombinase and a strand-displacing DNA polymerase to amplify DNA at a single, relatively low temperature (usually 37- 42°C). This isothermal property allows for rapid, portable DNA amplification, making it suitable for a variety of applications, including pathogen detection, genetic testing, and food safety analysis (Craw and Balachandran, 2012). Another advantage is that RPA requires minimal sample preparation and can amplify as few as 1–10 copies of the DNA target in less than 20 minutes. Today, RPA is used to detect a wide range of pathogens in human and veterinary medicine, agriculture, and food safety (Jinek et al., 2012; Lobato and O’Sullivan, 2018). Jinek et al. (2012) published an article stating that an adaptive immune system in prokaryotes called clustered regularly interspaced short palindromic repeats (CRISPR) can resist attack by foreign mobile genetic elements (MGEs) (Jinek et al., 2012). The CRISPR-Cas systems are classified into two classes (Classes 1 and 2). The Class 2 CRISPR-Cas systems assemble a ribonucleoprotein complex, consisting of a crRNA and a Cas protein.crRNA contains information for a specific DNA sequence. The Cas12a found in certain bacteria and archaea acts as molecular scissors to cut DNA. Since Cas12a has an endonuclease domain, it can be programmed via guide CRISPR RNA (crRNA) to target and cleave specific DNA sequences (Bhattacharya et al., 2024). Now, the RPA preamplification has been combined with CRISPR/Cas12a to develop nucleic acid diagnostic tools. Since the CRISPR/Cas12a reaction cleaves the reporter molecule, releasing a fluorescent signal, the signal can be easily visualized using a fluorescence detector or even a UV Illuminator. Based on this characteristic, portable detection instruments have been developed for on-site use (Li et al., 2024).

**Figure 5:**
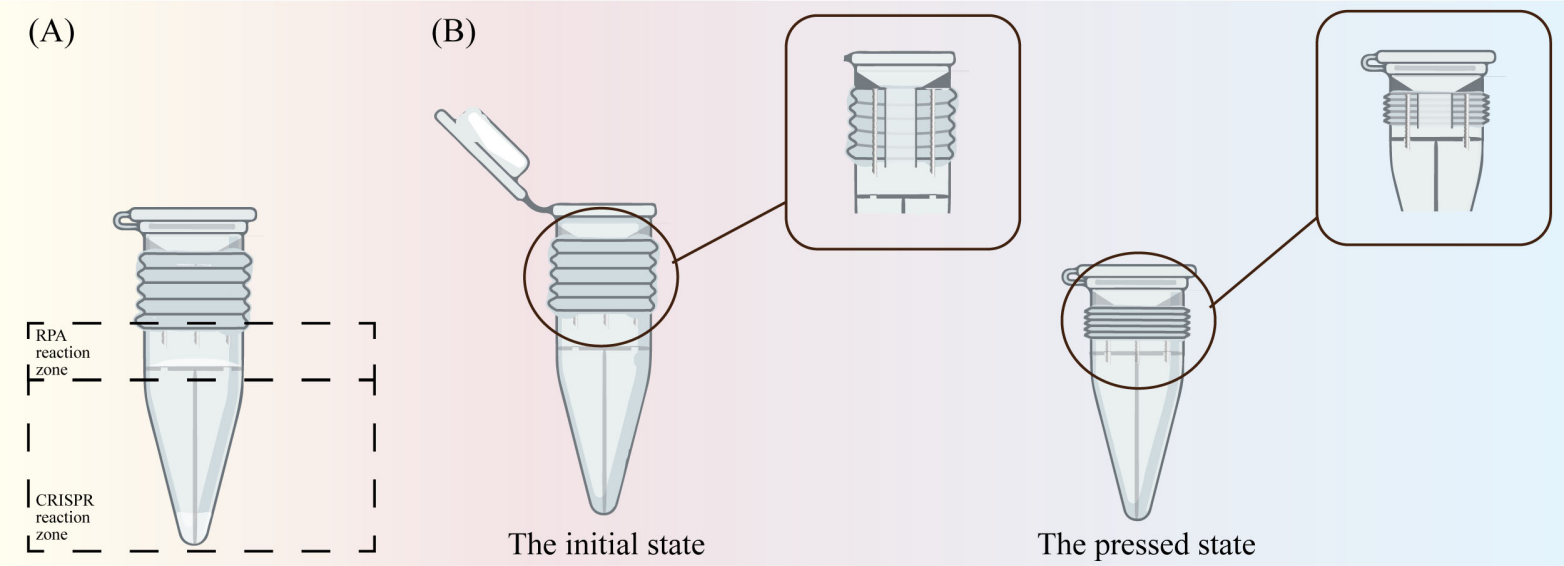
Design of integrated thermostatic reaction tube. **(A)** The initial state of the integrated thermostatic reaction tube **(B)** The press state of the integrated thermostatic reaction tube.

In this study, the RPA and CRISPR-Cas12a reactions were successfully integrated into a single tube under isothermal conditions for the detection of DVBV, BCoV, BRV, and ETEC from calf fecal samples and the signal amplification of fluorescence detection(**Figure 5**). Except for sample addition, the entire reaction was performed in a sealed tube, thereby avoiding contamination. The tube comprises two layers: the upper layer contains freeze-dried RPA reagent, while the lower layer features four independent compartments, each housing a CRISPR reaction system targeting distinct pathogens. During testing, after adding the sample and completing the RPA reaction in the upper layer, the tube is pressed to puncture the central membrane, allowing the RPA product to flow into each CRISPR compartment in the lower layer. Following reaction completion, the positive or negative of each pathogen is determined by assessing the fluorescent signal. Moreover, this device is capable of performing RPA-CRISPR/Cas12a detection, integrates amplification, cutting, and fluorescence detection into a compact and portable device, making it more suitable for on-site detection. The detection limits of BRV and BCoV were 16.4 copies/μL and 18.2 copies/μL, respectively (Meng et al., 2023). By comparing the results of the two detection methods, this study found that the RPA-CRISPR/Cas12a detection method can detect pathogen DNA as low as 10 copies/μL.

To determine whether the cause is due to experimental error, we will increase the number of tested samples in future studies while keeping all experimental conditions unchanged to improve the confidence and statistical significance of real-time qPCR results and compare them with the RPA-CRISPR/Cas12a detection results. We also noticed that in RPA-CRISPR/Cas12a assays, fluorescence intensity is measured in arbitrary units (a.u.). These units are not standardized across instruments or assays but are specific to the experimental setup. Intensity values are used to determine the presence and concentration of target DNA, with higher values indicating stronger signals. Therefore, in this study, the intensities of different samples can only be compared in consideration of the same instrument under the same conditions. Compared with real-time PCR, the concordance rate of RPA-CRISPR/Cas12a detection was 100% for BCoV, BRV, and ETEC, and 82% for BVDV. The detection results of the two methods were the same, except that the positive rate of RPA-CRISPR/Cas12a in detecting BVDV was slightly lower than that of real-time qPCR. Our result of detecting BVDV using the CRISPR/Cas12a assay was 15.3%, which is almost consistent with the results of other researchers. Wang et al. (2024) reported that BVDV was detected in 112 RT-PCR anal swab or nasal swab samples by RPA-CRISPR/Cas12a detection and. The positive rates detected by the two methods were highly consistent, at 14.3% (16/112) (Wang et al., 2024). We noted that the positivity rate of the detection is also related to the current site epidemiology. For example, in another recent study, we found that 78.1% of farms were positive for calf diarrhea virus infection, and the co-infection rate on farms was 29.3%, with BRV and BCoV being the most common co-infection combination. There remains considerable room for improvement in this study. In future work, we will expand the sample size and include additional clinical and field specimens to comprehensively evaluate the method’s applicability and stability in real-world settings. Moreover, direct RNA detection based on the Cas13 protein has recently attracted increasing attention; owing to its high sensitivity and specificity, it offers a promising strategy for multiplex detection of RNA viruses (Wang et al., 2025). Subsequent studies should consider integrating our approach with Cas13-based assays or conducting direct comparative evaluations to explore solutions that enable higher throughput, greater generalizability, and enhanced field deployability for surveillance of animal-derived viruses.

In summary, we recently developed and demonstrated an RPA-CRISPR/Cas12a assay for the simultaneous detection of BVDV, BCoV, BRV, and ETEC pathogens in fecal diarrhea samples from calves. To the best of our knowledge, this is the first report on the detection of four diarrheal pathogens using the RPA-CRISPR/Cas12a assay. Due to its sensitivity, specificity, simplicity, and rapidity, the RPA-CRISPR/Cas12a assay is very useful for on-site diagnosis of cattle diarrhea pathogens.

## Data Availability

The original contributions presented in the study are included in the article/**Supplementary Material**. Further inquiries can be directed to the corresponding author.
