# Supplementary material for: Sensitive, specific, and rapid on-site detection of calf diarrhea pathogens using the RPA-CRISPR/Cas 12a assay
Source: Front Cell Infect Microbiol. 2026 Feb 2;16:1734185. doi: 10.3389/fcimb.2026.1734185 (PMC12907351; doi:10.3389/fcimb.2026.1734185)
Supplement: Supplementary file 1 [file Table1.docx]

Supplementary Material

| RPA-CRISPR/Cas 12a Assay | | | | | | | | | | | | | | | | | | | | |
| --- | --- | --- | --- | --- | --- | --- | --- | --- | --- | --- | --- | --- | --- | --- | --- | --- | --- | --- | --- | --- |
| Sample Number | **1** | **2** | **3** | **4** | **5** | **6** | **7** | **8** | **9** | **10** | **11** | **12** | **13** | **14** | **15** | **16** | **17** | **18** | **19** | **20** |
| Mean gray value(GV) | 23.6 | 25 | 24.6 | 24.1 | 29.5 | 87.7 | 25.9 | 21.8 | 110.2 | 27 | 29 | 25.1 | 23.0 | 62.8 | 23.7 | 24.1 | 21.3 | 99.2 | 26.2 | 23.8 |
| Result | — | — | — | — | — | **＋** | — | — | **＋** | — | — | — | — | **＋** | — | — | — | **＋** | — | — |
|  | **21** | **22** | **23** | **24** | **25** | **26** | **27** | **28** | **29** | **30** | **31** | **32** | **33** | **34** | **35** | **36** | **37** | **38** | **39** | **40** |
|  | 27.2 | 26.5 | 23.3 | 22.2 | 23.7 | 64 | 28.2 | 67.5 | 25.7 | 28.6 | 27.9 | 27.5 | 24.8 | 40.7 | 33.2 | 29.1 | 28.7 | 54.2 | 24.7 | 137.6 |
|  | — | — | — | — | — | **＋** | — | **＋** | — | — | — | — | — | **＋** | — | — | — | **＋** | — | **＋** |
|  | **41** | **42** | **43** | **44** | **45** | **46** | **47** | **48** | **49** | **50** | **51** | **52** | **53** | **54** | **55** | **56** | **57** | **58** | **59** | **NC** |
|  | 34.3 | 25.2 | 21.2 | 20.2 | 27.1 | 22.5 | 23.4 | 21.3 | 25.9 | 22.8 | 28.5 | 29.8 | 24.3 | 25.2 | 22 | 29 | 22.5 | 28.1 | 19.2 | 20.6 |
|  | — | — | — | — | — | — | — | — | — | — | — | — | — | — | — | — | — | — | — | — |

**Table S1. ImageJ Analysis of Mean Grayscale Values for RPA-CRISPR/Cas12a assay BVDV and Result Judgment**

**Table S2. ImageJ Analysis of Mean Grayscale Values for RPA-CRISPR/Cas12a assay BCoV and Result Judgment**

| RPA-CRISPR/Cas 12a Assay | | | | | | | | | | | | | | | | | | | | |
| --- | --- | --- | --- | --- | --- | --- | --- | --- | --- | --- | --- | --- | --- | --- | --- | --- | --- | --- | --- | --- |
| Sample Number | **1** | **2** | **3** | **4** | **5** | **6** | **7** | **8** | **9** | **10** | **11** | **12** | **13** | **14** | **15** | **16** | **17** | **18** | **19** | **20** |
| Mean gray value(GV) | 23.6 | 23.4 | 23.3 | 22.6 | 20.2 | 25.1 | 25.9 | 24.4 | 23.7 | 92.2 | 21.5 | 21.8 | 22.4 | 20.8 | 25.8 | 25.1 | 22.1 | 19.2 | 18.2 | 15.03 |
| Result | — | — | — | — | — | — | — | — | — | **＋** | — | — | — | — | — | — | — | — | — | — |
|  | **21** | **22** | **23** | **24** | **25** | **26** | **27** | **28** | **29** | **30** | **31** | **32** | **33** | **34** | **35** | **36** | **37** | **38** | **39** | **40** |
|  | 23 | 20.7 | 22.0 | 25.1 | 23.3 | 20.4 | 20 | 14.5 | 16.9 | 23.7 | 23.3 | 21.7 | 23.7 | 20.1 | 18 | 14.1 | 26.4 | 26.5 | 26.5 | 26.1 |
|  | — | — | — | — | — | — | — | — | — | — | — | — | — | — | — | — | — | — | — | — |
|  | **41** | **42** | **43** | **44** | **45** | **46** | **47** | **48** | **49** | **50** | **51** | **52** | **53** | **54** | **55** | **56** | **57** | **58** | **59** | **NC** |
|  | 24.3 | 25.2 | 21.2 | 20.2 | 27.1 | 22.5 | 23.4 | 21.3 | 25.9 | 22.8 | 28.5 | 29.8 | 24.3 | 25.2 | 22 | 26 | 22.5 | 28.1 | 19.2 | 20.6 |
|  | — | — | — | — | — | — | — | — | — | — | — | — | — | — | — | — | — | — | — | — |

**Table S3. ImageJ Analysis of Mean Grayscale Values for RPA-CRISPR/Cas12a assay BRV and Result Judgment**

| RPA-CRISPR/Cas 12a Assay | | | | | | | | | | | | | | | | | | | | |
| --- | --- | --- | --- | --- | --- | --- | --- | --- | --- | --- | --- | --- | --- | --- | --- | --- | --- | --- | --- | --- |
| Sample Number | **1** | **2** | **3** | **4** | **5** | **6** | **7** | **8** | **9** | **10** | **11** | **12** | **13** | **14** | **15** | **16** | **17** | **18** | **19** | **20** |
| Mean gray value(GV) | 42.7 | 26.7 | 27.3 | 26.8 | 27 | 40.7 | 26.6 | 25.6 | 237 | 20.7 | 20.3 | 25.5 | 119.6 | 86.1 | 65.3 | 136 | 124.4 | 21.1 | 20.6 | 20.5 |
| Result | **＋** | — | — | — | — | **＋** | — | — | **＋** | — | — | — | **＋** | **＋** | **＋** | **＋** | **＋** | — | — | — |
|  | **21** | **22** | **23** | **24** | **25** | **26** | **27** | **28** | **29** | **30** | **31** | **32** | **33** | **34** | **35** | **36** | **37** | **38** | **39** | **40** |
|  | 46 | 84.1 | 30.8 | 28.6 | 27.6 | 27.5 | 21.5 | 20.3 | 28.6 | 20.1 | 21.3 | 27.7 | 59.1 | 56.8 | 24.8 | 22.3 | 22.2 | 134 | 69.9 | 62.5 |
|  | **＋** | **＋** | — | — | — | — | — | — | — | — | — | — | **＋** | **＋** | — | — | — | **＋** | **＋** | **＋** |
|  | **41** | **42** | **43** | **44** | **45** | **46** | **47** | **48** | **49** | **50** | **51** | **52** | **53** | **54** | **55** | **56** | **57** | **58** | **59** | **NC** |
|  | 133.4 | 21.9 | 147.3 | 23.2 | 62.9 | 22.1 | 68.1 | 29.1 | 23 | 22.3 | 85.7 | 20.4 | 20.6 | 46 | 11 | 60.7 | 25.63 | 27.109 | 26.9 | 27.6 |
|  | **＋** | — | **＋** | — | **＋** | — | **＋** | — | — | — | **＋** | — | — | **＋** | — | **＋** | — | — | — | — |

**Table S4. ImageJ Analysis of Mean Grayscale Values for RPA-CRISPR/Cas12a assay ETEC and Result Judgment**

| RPA-CRISPR/Cas 12a Assay | | | | | | | | | | | | | | | | | | | | |
| --- | --- | --- | --- | --- | --- | --- | --- | --- | --- | --- | --- | --- | --- | --- | --- | --- | --- | --- | --- | --- |
| Sample Number | **1** | **2** | **3** | **4** | **5** | **6** | **7** | **8** | **9** | **10** | **11** | **12** | **13** | **14** | **15** | **16** | **17** | **18** | **19** | **20** |
| Mean gray value(GV) | 69.6 | 26.1 | 26.4 | 25.6 | 25.5 | 26.1 | 30 | 28.4 | 20.4 | 27.8 | 29.3 | 26.5 | 124.2 | 76.3 | 51.5 | 150.9 | 23.3 | 19.7 | 22 | 20.8 |
| Result | **＋** | — | — | — | — | — | — | — | — | — | — | — | **＋** | **＋** | **＋** | **＋** | — | — | — | — |
|  | **21** | **22** | **23** | **24** | **25** | **26** | **27** | **28** | **29** | **30** | **31** | **32** | **33** | **34** | **35** | **36** | **37** | **38** | **39** | **40** |
|  | 36.6 | 19.2 | 20.5 | 134.2 | 99.1 | 20.7 | 19 | 22 | 21 | 14.7 | 20.5 | 20.8 | 23.1 | 25.2 | 89.8 | 23.2 | 23.1 | 25.3 | 29 | 29.1 |
|  | **＋** | — | — | **＋** | **＋** | — | — | — | — | — | — | — | — | — | **＋** | — | — | — | — | — |
|  | **41** | **42** | **43** | **44** | **45** | **46** | **47** | **48** | **49** | **50** | **51** | **52** | **53** | **54** | **55** | **56** | **57** | **58** | **59** | **NC** |
|  | 20.9 | 24 | 22.9 | 20.8 | 25.6 | 34.4 | 22.2 | 21.2 | 23.2 | 85.6 | 25.0 | 108.5 | 18.1 | 70.5 | 16.8 | 71.1 | 39.5 | 93.7 | 86.6 | 21.5 |
|  | — | — | — | — | — | **＋** | — | — | — | **＋** | — | **＋** | — | **＋** | — | **＋** | **＋** | **＋** | **＋** | — |

**＋ : test result is positive ➖ : negative control**

# Table 5. **Diagnostic Evaluation of the RPA‑CRISPR/Cas12a Assay**

|  | BVDV | BCoV | BRV | ETEC |
| --- | --- | --- | --- | --- |
| Sensitivity (%) | 81.8 | 100 | 100 | 100 |
| 95% CI | 52.3–95.1 % | 59.0–100.0 % | 88.1–100.0 % | 84.7–100.0 % |
| Specificity (%) | 100 | 100 | 100 | 100 |
| 95% CI | 92.6–100.0 % | 93.8–100.0 % | 90.5–100.0 % | 93.0–100.0 % |
| PPV(%) | 100 | 100 | 100 | 100 |
| 95% CI | 70.0–100.0 % | 59.0–100.0 % | 88.1–100.0 % | 84.7–100.0 % |
| NPV(%) | 100 | 100 | 100 | 100 |
| 95% CI | 86.3–99.0 % | 93.8–100.0 % | 90.5–100.0 % | 93.0–100.0 % |
| kappa | 0.88 | 1.00 | 1.00 | 1.00 |

# Fig. S1.The conserved sequence alignment of four viruses

1. BVDV 5'-UTR region；（B）BCoV N region；（C）BRV VP7 region；ETEC STa region

(A)


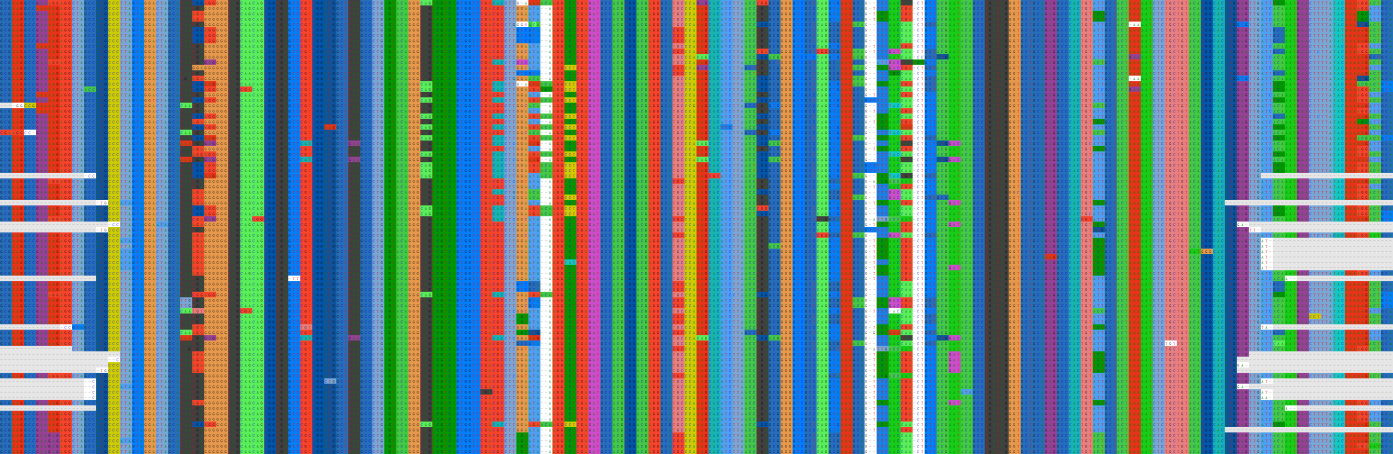


(B)


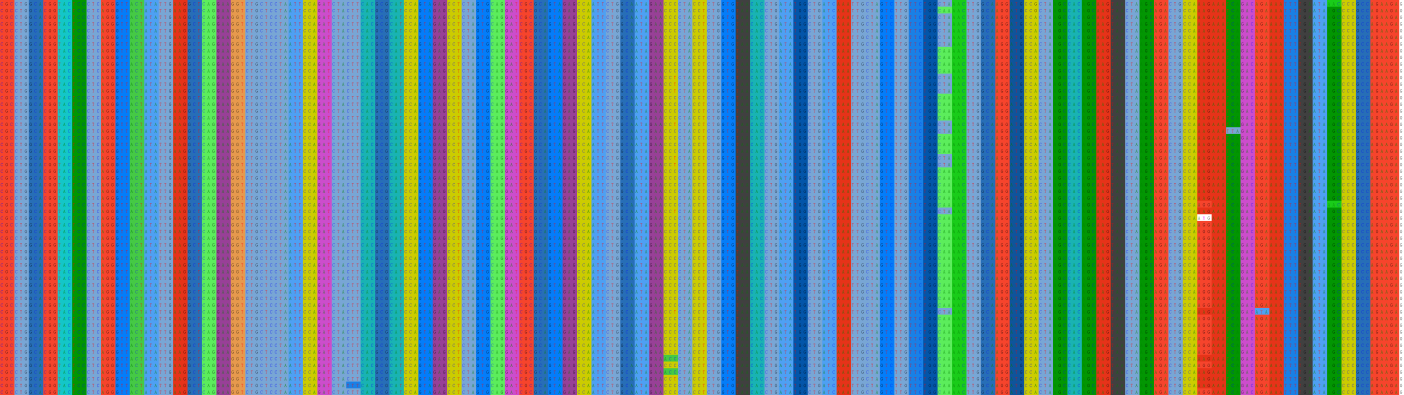


(C)

**
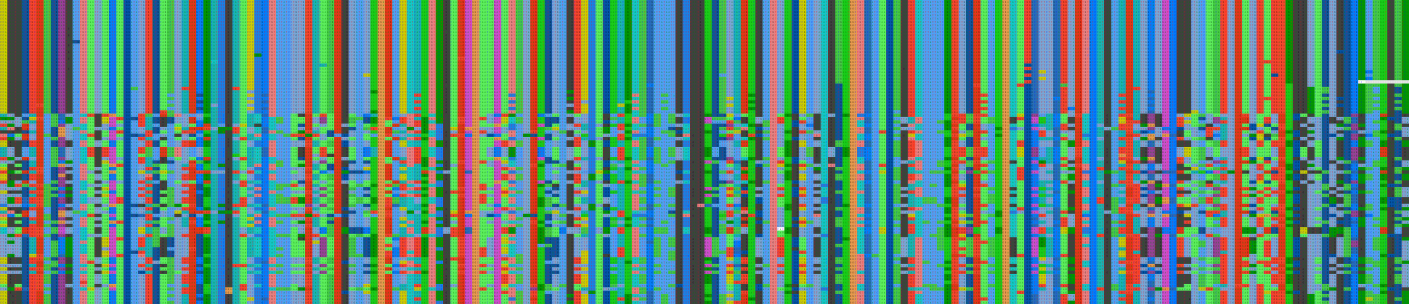
**

(D)


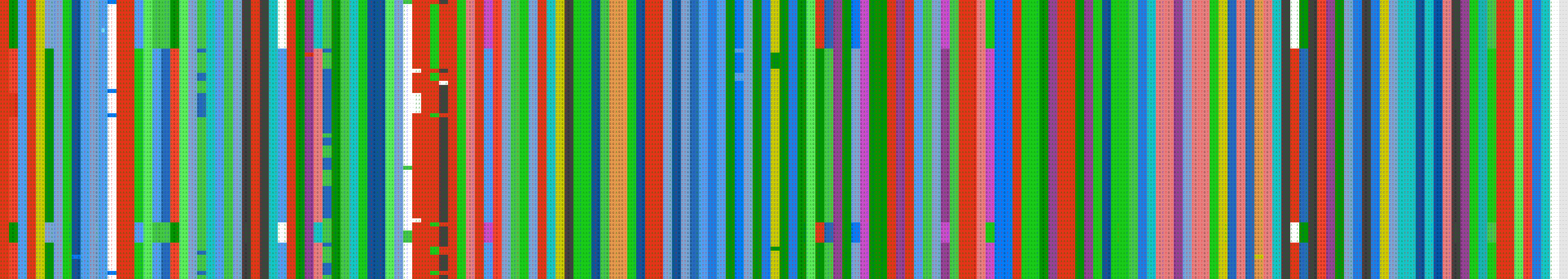


**Fig.S2. Screening of primers for amplification of four pathogens**

（A）BVDV；（B）BCoV；（C）BRV；(D)ETEC


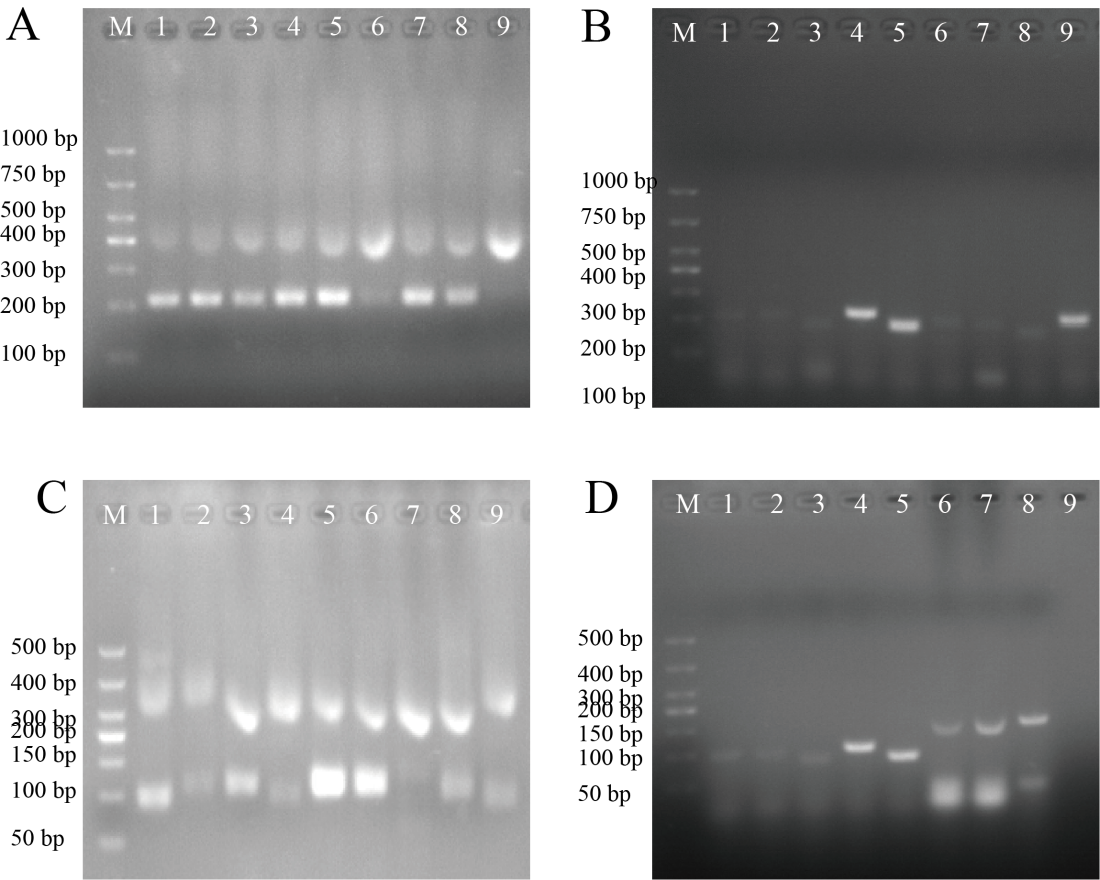


**Fig. S3 CRISPR Cas12a systems optimization.**10ul reaction mixture consists of 1 ul NEB Buffer 2.1, 0.5 ul LbaCas12a, 0.5ul ssDNA Reporter, 0.5ul crRNA, 6.5 ul DEPC-treated water, and 2 ul RPA products,other volumetric reaction systems were scaled proportionally, but the template quantity remained unchanged, with the volume adjusted using DEPC-treated water.


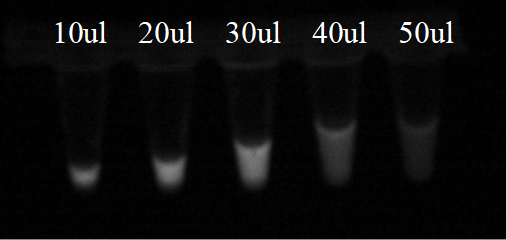


**Fig.S4. Real-time fluorescence detection of RPA-CRISPR/Cas12a crRNA.** (A) Real-time fluorescence detection of BVDV crRNA. (B) Real-time fluorescence detection of BCoV crRNA. (C) Real-time fluorescence detection of BRV crRNA. (D) Real-time fluorescence detection of BVDV crRNA.

**
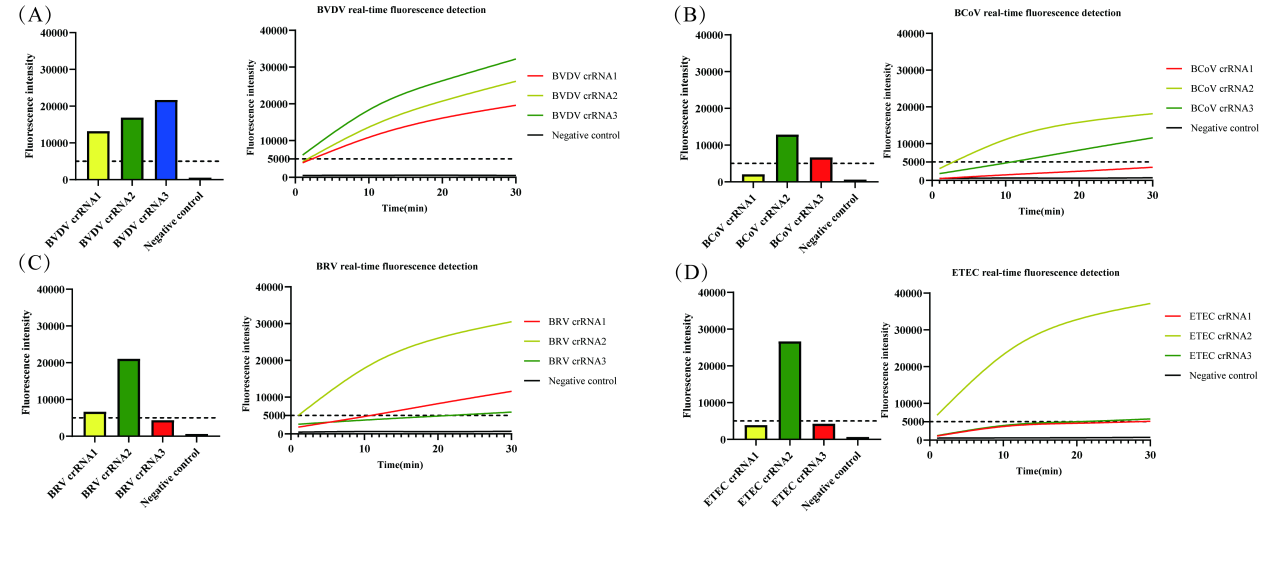
**

**Fig.S5 Real-time fluorescence detection of RPA-CRISPR/Cas12a ssDNA reporter concentration optimization.** (A) Real-time fluorescence detection of BVDV ssDNA reporter concentration optimization. (B) Real-time fluorescence detection of BCoV ssDNA reporter concentration optimization. (C) Real-time fluorescence detection of BRV ssDNA reporter concentration optimization. (D) Real-time fluorescence detection of ETEC ssDNA reporter concentration optimization.

**
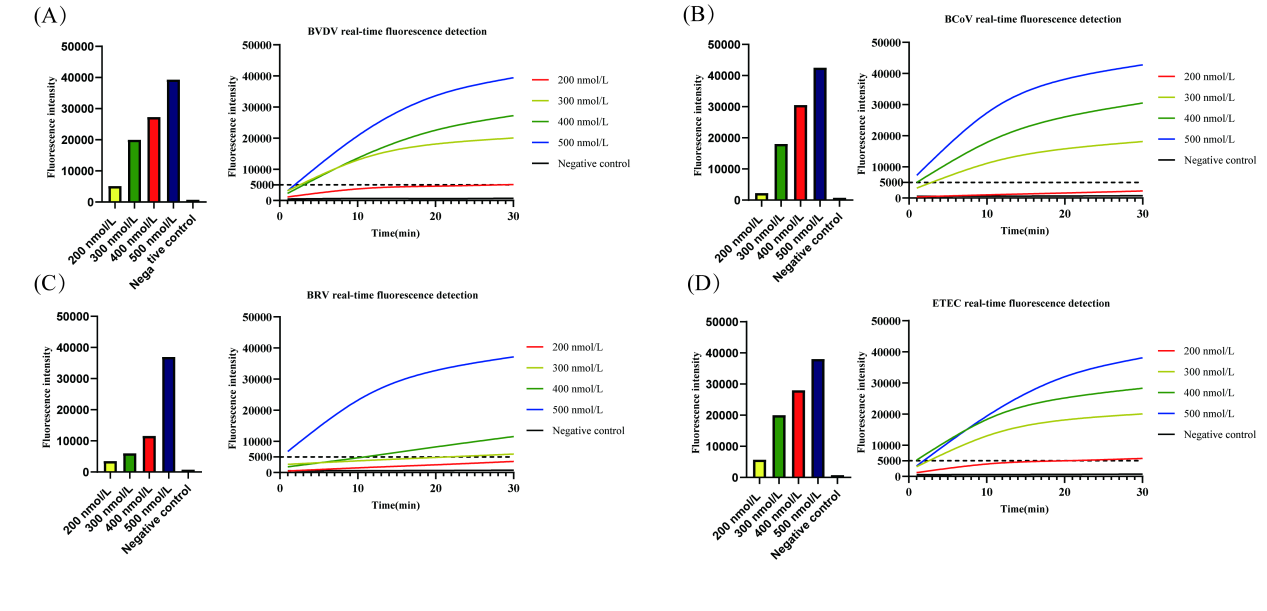
**

**Fig.S6 Real-time fluorescence results of crRNAN and Cas12a proteins.**The results of the of crRNA vs. Cas12a concentration for BVDV (A), BCoV (B), BRV (C), and ETEC (D) were presented using fluorescence, and the Maximum fluorescence intensity were selected as the optimal conditions.

**
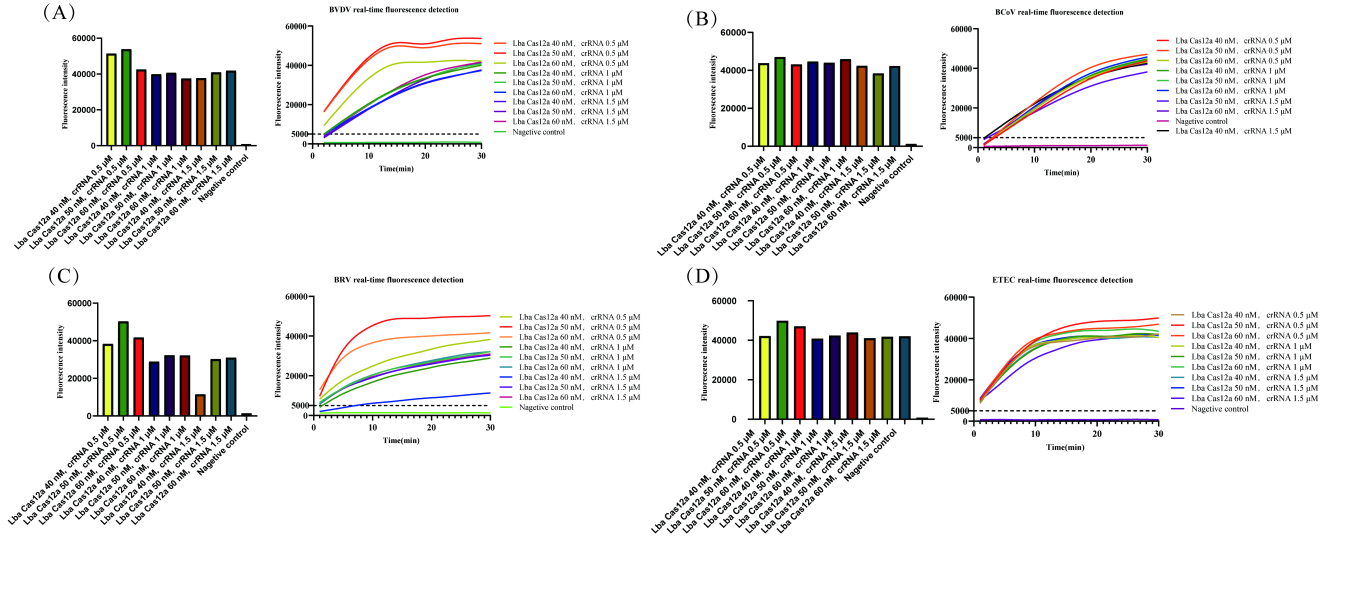
**

**Fig.S7 Experiments on different RPA-CRISPR/Cas12a reaction systems.**The results of detecting six different RPA-CRISPR/Cas12a reaction systems of BVDV (A), BCoV (B), BRV (C) and ETEC (D) by under UV light, under blue light and real-time fluorescence detection methods.

(D)
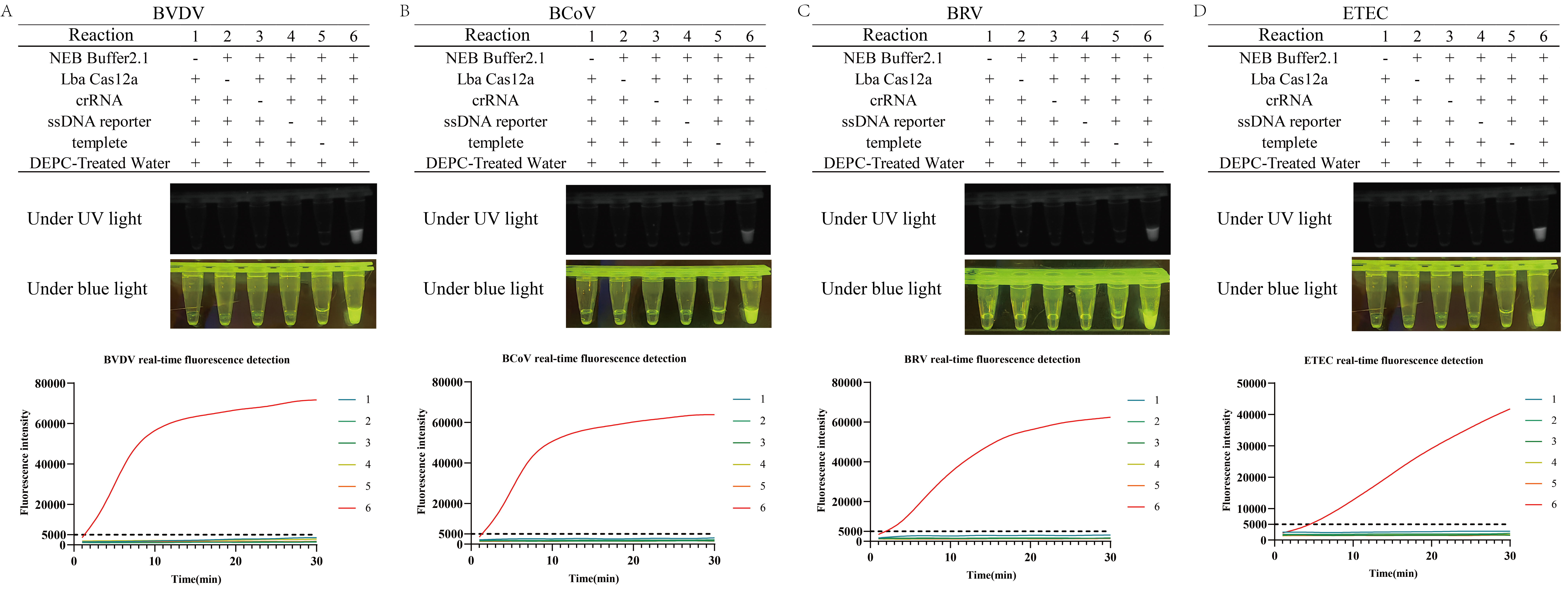


(C)
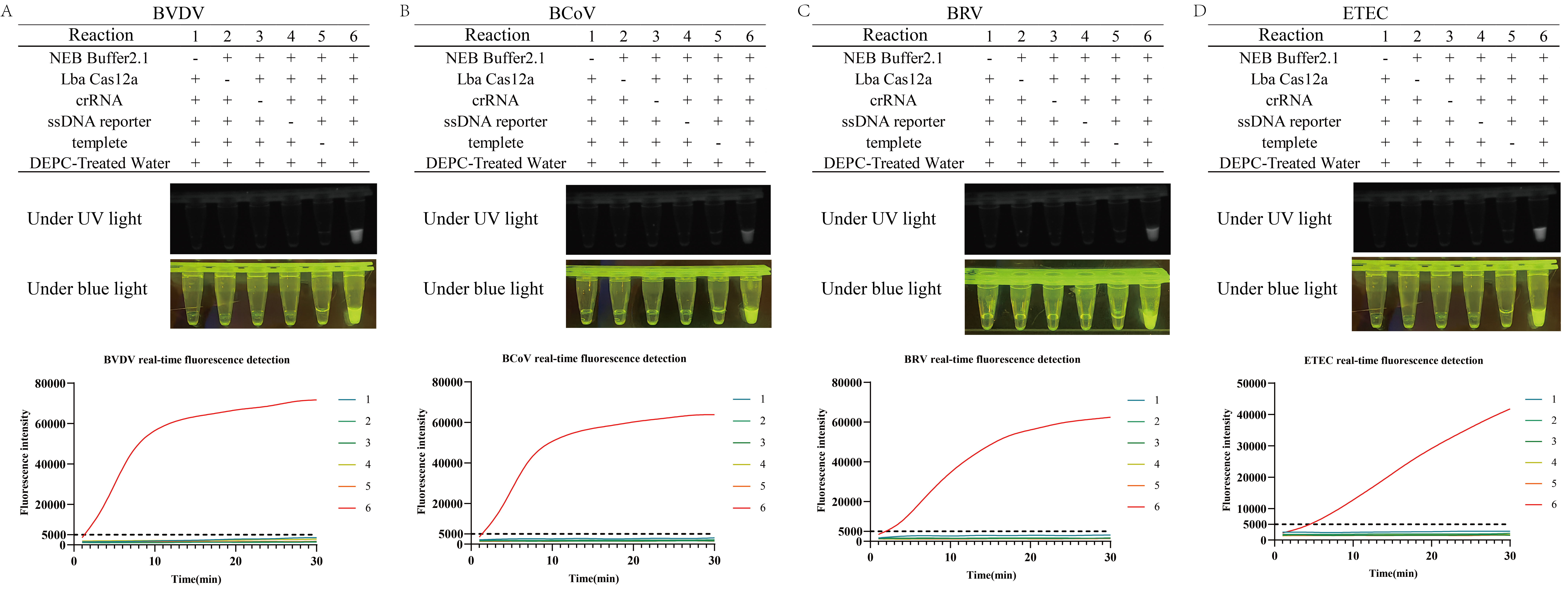


(B)
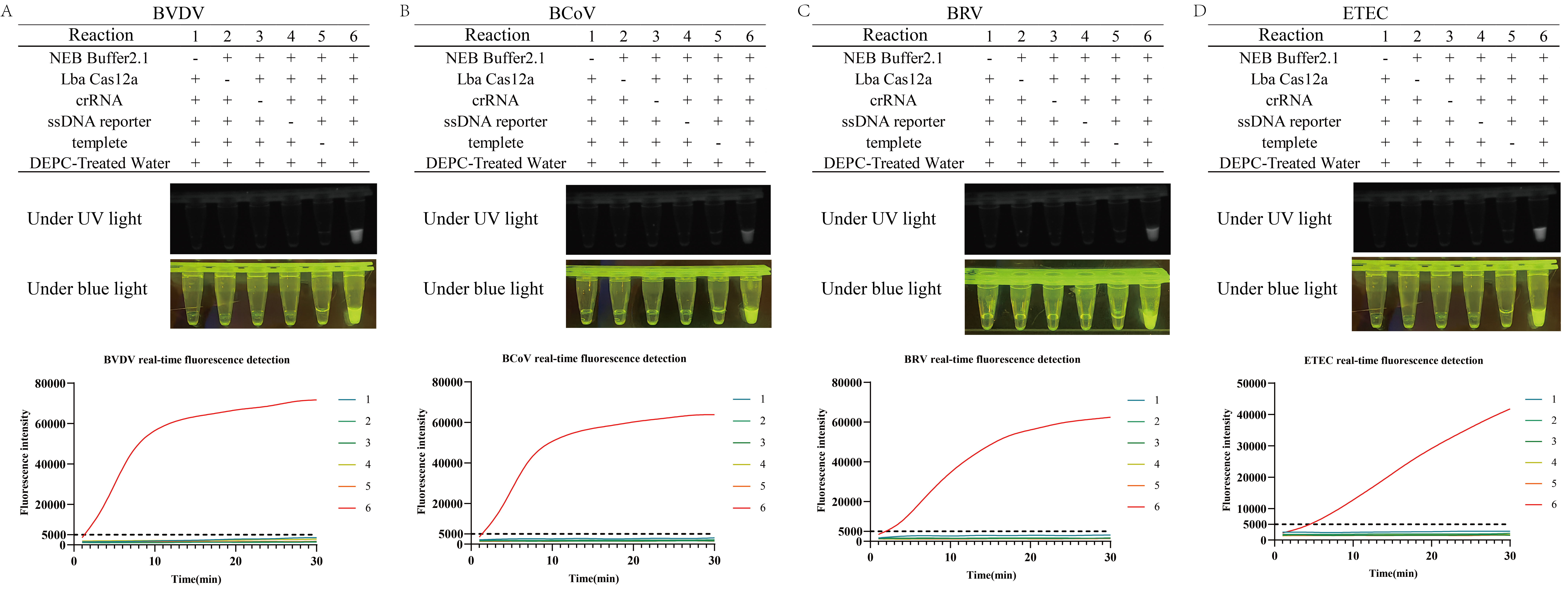


(A)
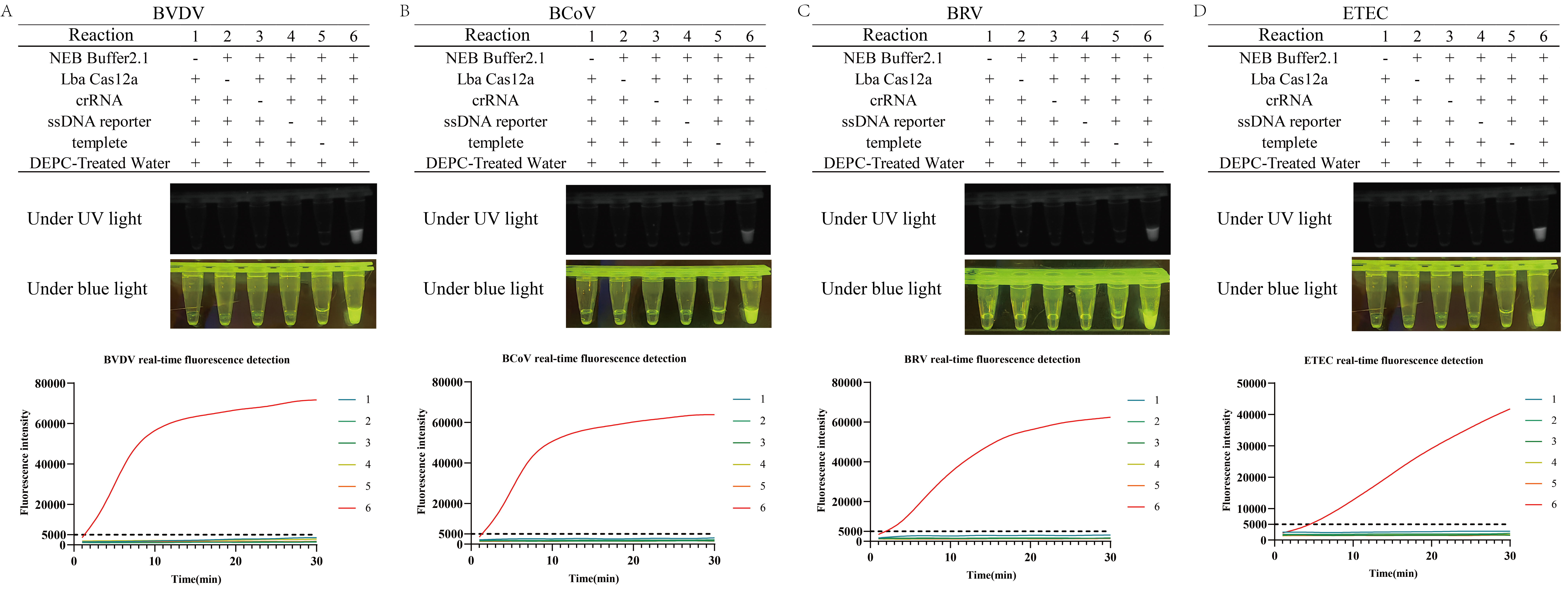


**
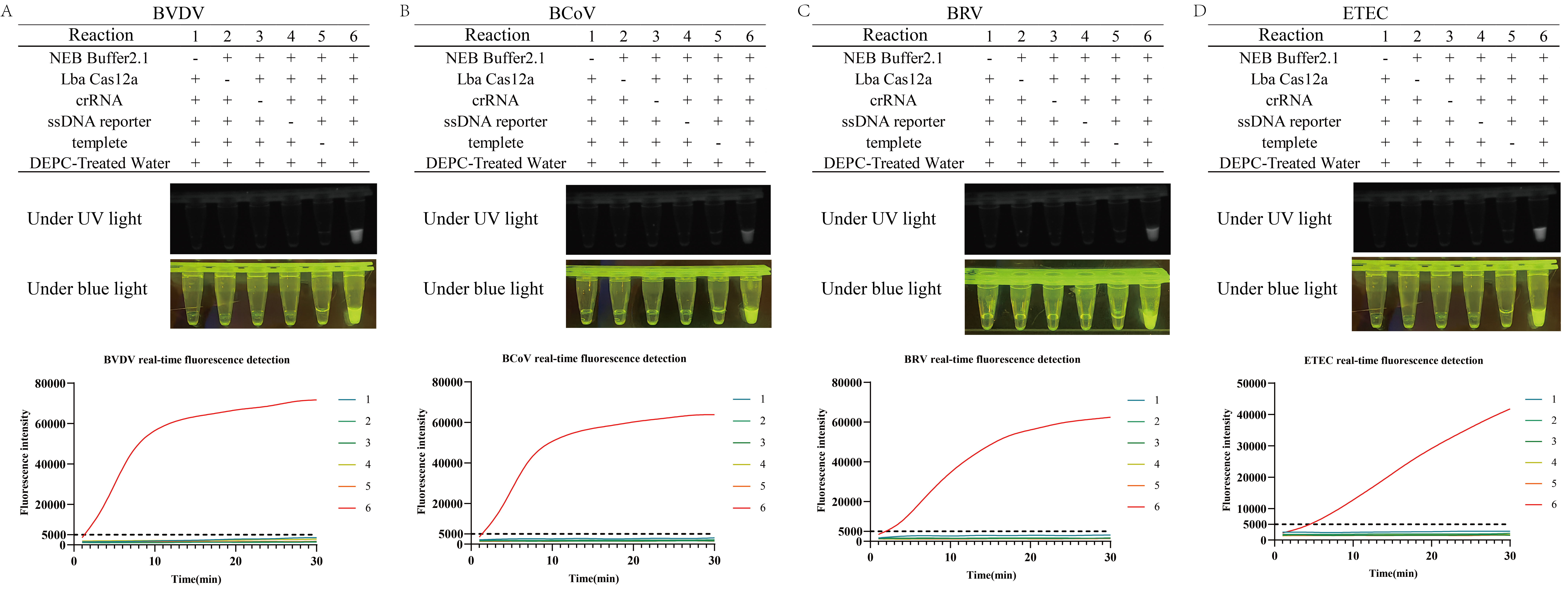
**

**Fig.S8 Real‑time fluorescence kinetics demonstrating the Sensitivity of RPA‑CRISPR/Cas12a.**The fluorescence kinetics demonstrating of (A) BVDV, (B) BCoV, (C) BRV, and (D) ETEC increased from 10 copies/μL to 105 copies/μL.

**
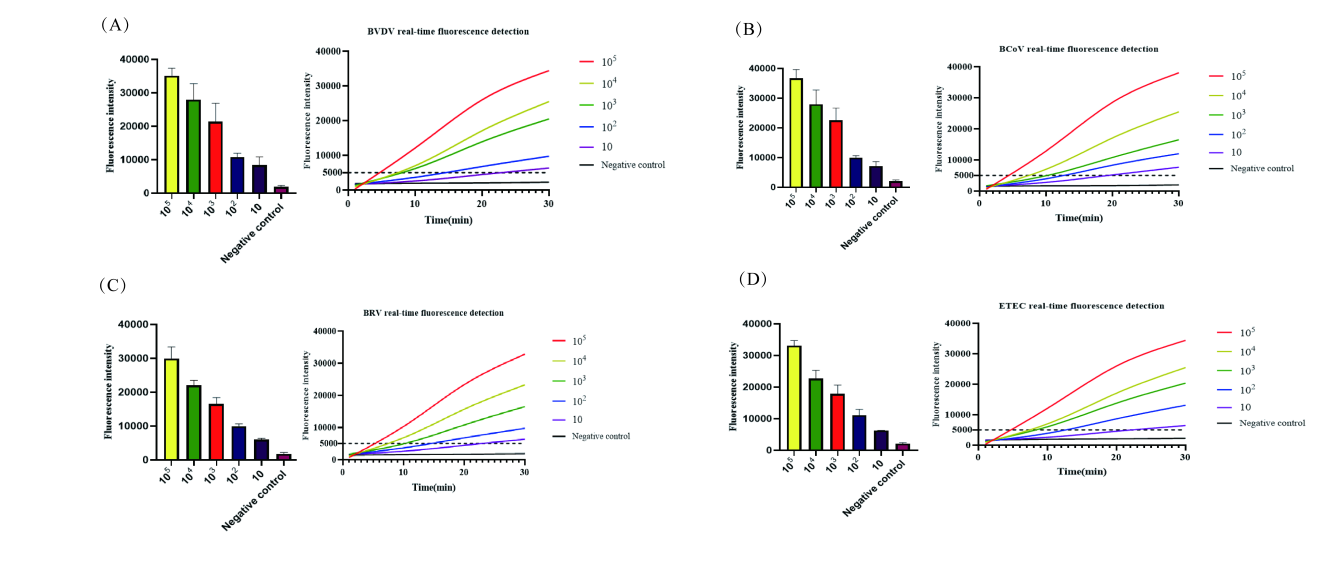
**

**Fig.9 The fluorescence kinetic for Specificity of RPA-CRISPR/Cas12a fluorescence detection.**

**
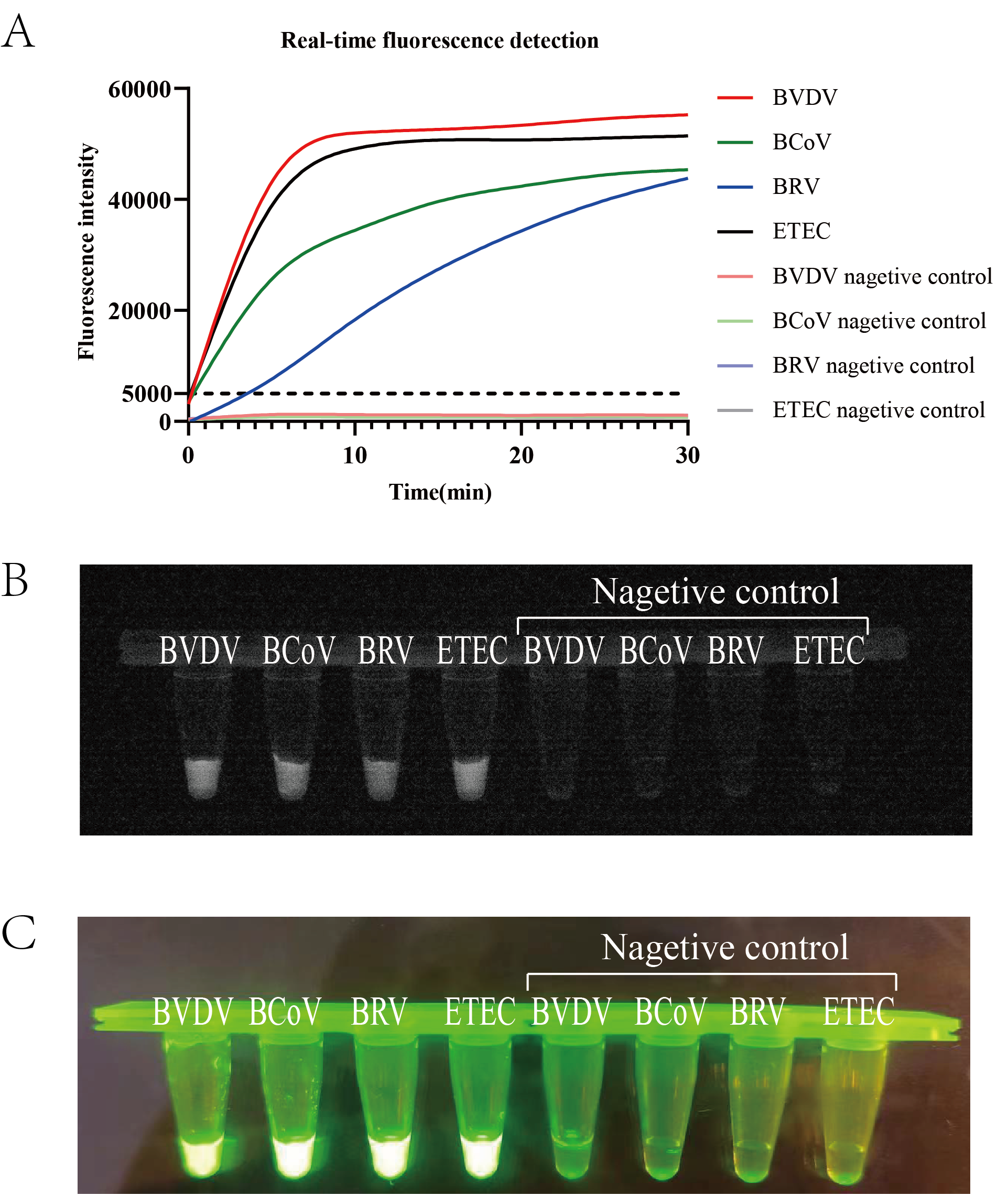
**

**Fig.S10. Real‑time Fluorescence Kinetics for Specificity Testing of RPA‑CRISPR/Cas12a.**(A) First column contains the plasmid of BVDV DNA, and the other columns are negative controls. (B) First column contains the plasmid of BCov DNA, and the other columns are negative controls. (C) First column contains the plasmid of BRV DNA, and the other columns are negative controls. (D) First column contains the plasmid of ETEC DNA, and the other columns are negative controls.

**
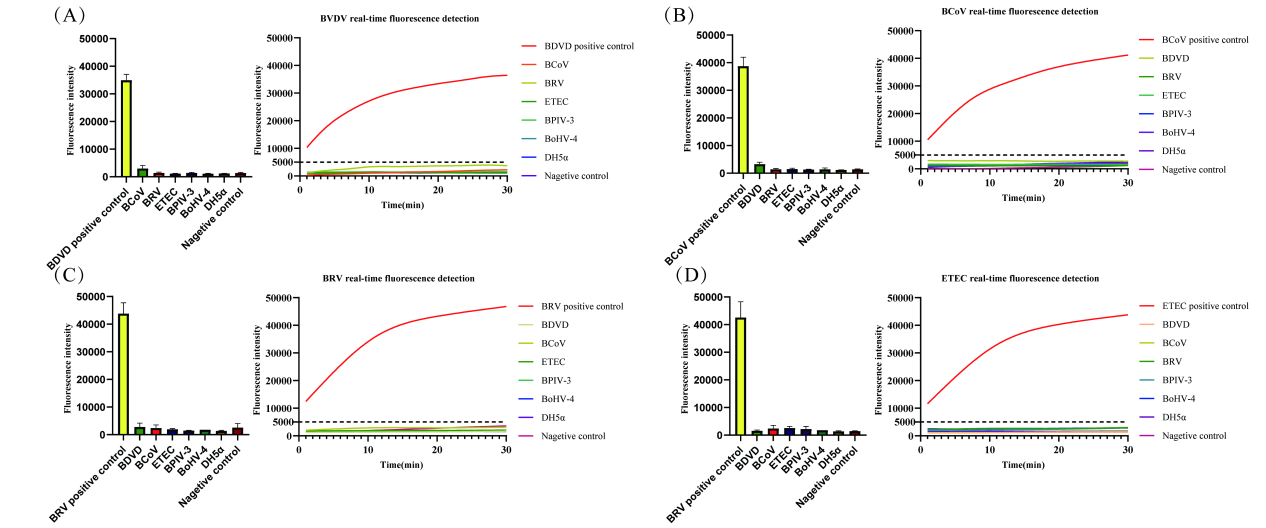
**

**Fig.S11 RPA-CRISPR/Cas12a sample detection diagram.** The detection results of RPA-CRISPR/Cas12a BVDV (A), BCoV (B), BRV (C) and ETEC (D) samples were observed by UV light and blue light.

**
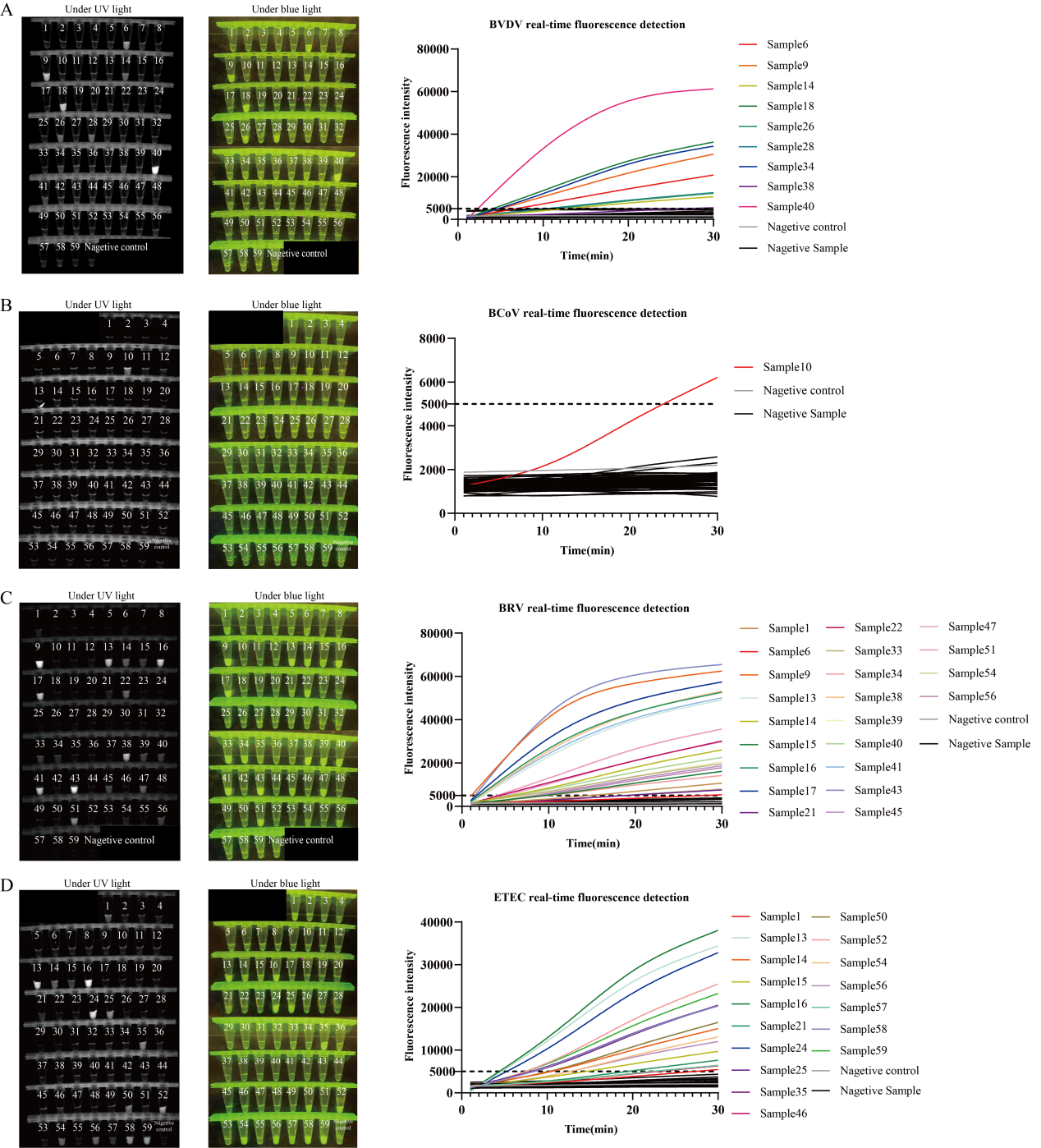
**

**Fig. S12.qPCR sample detection diagram.** (A) BVDV, (B) BCoV, (C) BRV and (D)ETEC sample detection results.


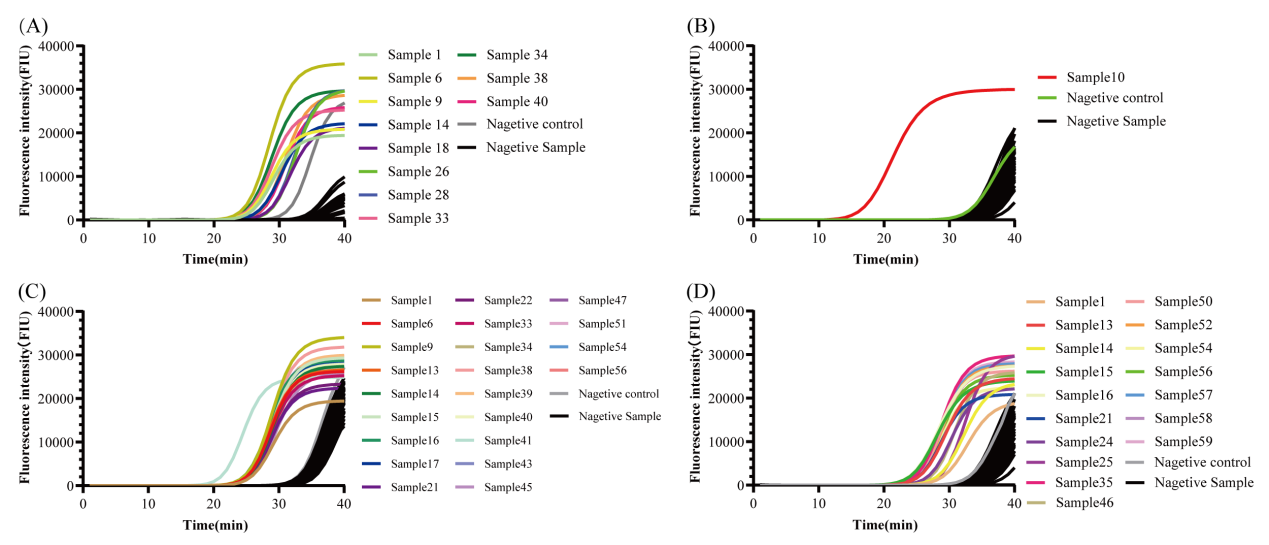


**Fig. S13.Positive test results of PRA-CRISPR/Cas12a assay and Real-time qPCR assay**

**
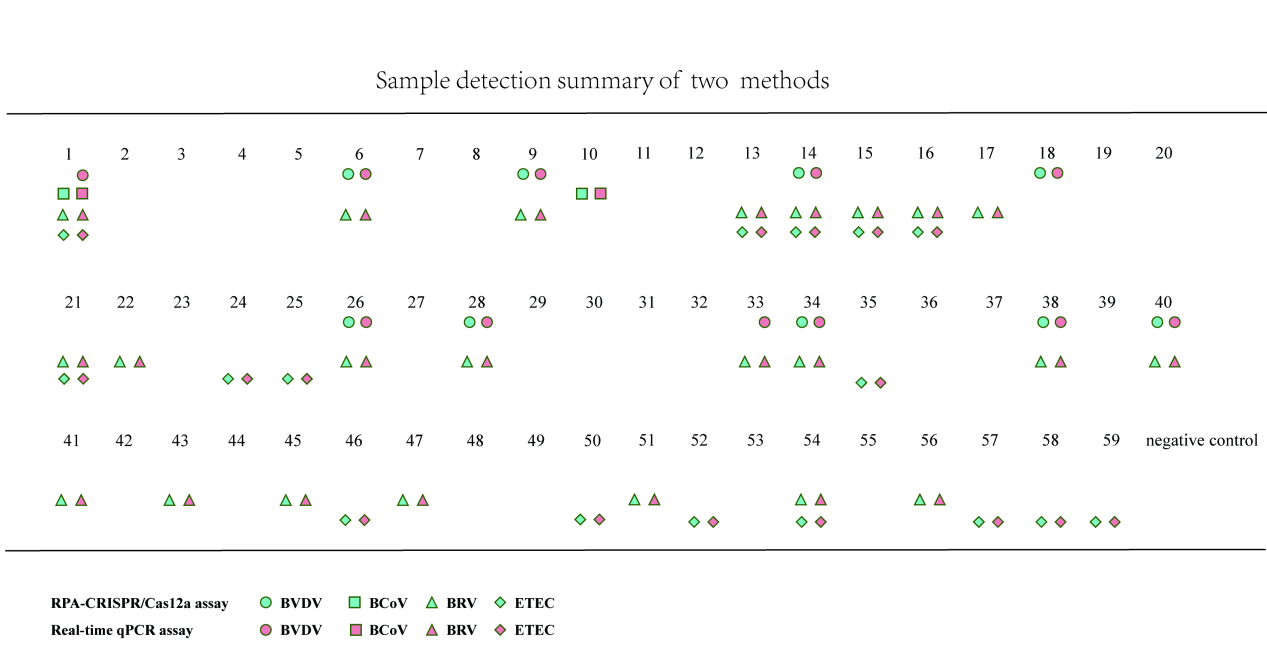
**

**Fig. S14 Figure 1.uncropped Gels image**

**
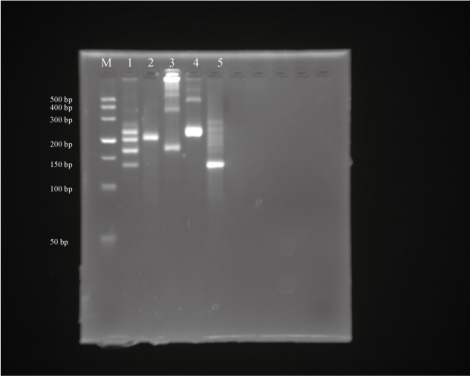
**

**Fig. S15 Fig. S1. uncropped Gels image**

**
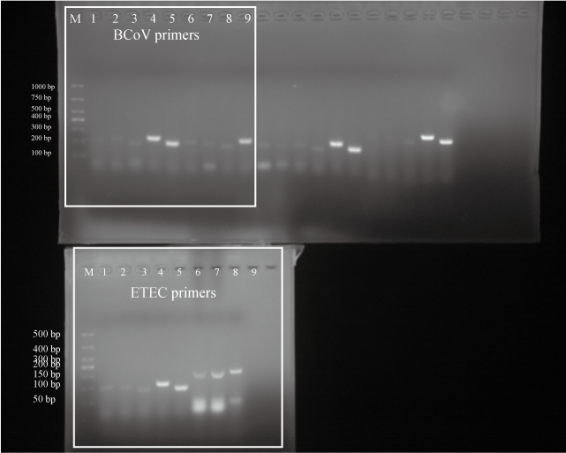

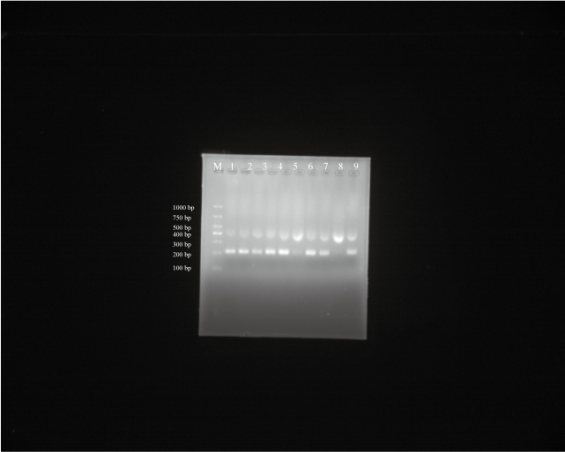
**

BVDV primers

**
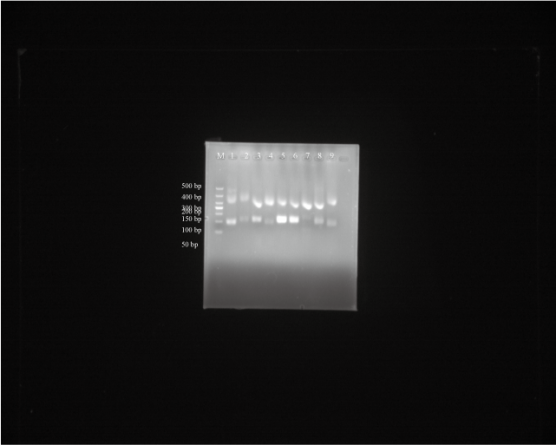
**

BRV primers
